# Structures of human γδ T cell receptor–CD3 complex

**DOI:** 10.1038/s41586-024-07439-4

**Published:** 2024-04-24

**Authors:** Weizhi Xin, Bangdong Huang, Ximin Chi, Yuehua Liu, Mengjiao Xu, Yuanyuan Zhang, Xu Li, Qiang Su, Qiang Zhou

**Affiliations:** 1 Research Center for Industries of the Future, Center for Infectious Disease Research, Zhejiang Key Laboratory of Structural Biology, School of Life Sciences, Westlake University, Hangzhou, China; 2 Westlake Laboratory of Life Sciences and Biomedicine, Hangzhou, China; 3 Institute of Biology, Westlake Institute for Advanced Study, Hangzhou, China; 4 Present Address: State Key Laboratory of Cellular Stress Biology, Innovation Center for Cell Signaling Network, School of Life Science, Xiamen University, Xiamen, China

**Keywords:** Cryoelectron microscopy, Antigen presentation

## Abstract

Gamma delta (γδ) T cells, a unique T cell subgroup, are crucial in various immune responses and immunopathology^1–3^. The γδ T cell receptor (TCR), which is generated by γδ T cells, recognizes a diverse range of antigens independently of the major histocompatibility complex^2^. The γδ TCR associates with CD3 subunits, initiating T cell activation and holding great potential in immunotherapy^4^. Here we report the structures of two prototypical human Vγ9Vδ2 and Vγ5Vδ1 TCR–CD3 complexes^5,6^, revealing two distinct assembly mechanisms that depend on Vγ usage. The Vγ9Vδ2 TCR–CD3 complex is monomeric, with considerable conformational flexibility in the TCRγ–TCRδ extracellular domain and connecting peptides. The length of the connecting peptides regulates the ligand association and T cell activation. A cholesterol-like molecule wedges into the transmembrane region, exerting an inhibitory role in TCR signalling. The Vγ5Vδ1 TCR–CD3 complex displays a dimeric architecture, whereby two protomers nestle back to back through the Vγ5 domains of the TCR extracellular domains. Our biochemical and biophysical assays further corroborate the dimeric structure. Importantly, the dimeric form of the Vγ5Vδ1 TCR is essential for T cell activation. These findings reveal organizing principles of the γδ TCR–CD3 complex, providing insights into the unique properties of γδ TCR and facilitating immunotherapeutic interventions.

## Abstract (web summary)

The assembly of the γδ TCR depends on Vγ usage.

## Main

T cells are critical components of the vertebrate immune system, defending against invading pathogens and malignant cells. Human T lymphocytes are classified into two lineages, αβ and γδ T cells, based on their expression of either αβ or γδ T cell receptors (TCRs)^1,2^. In contrast to conventional αβ T cells, γδ T cells exhibit both adaptive and innate immune properties, constituting a unique lymphocyte population^3,7^. γδ T cells provide broad protective immune responses against various infectious and sterile stresses, including cytomegalovirus and *Mycobacterium tuberculosis* infections, tumour growth and tissue dysregulation^2,3,8^.

The γδ TCR is a heterodimer comprising TCRγ and TCRδ chains, discovered in the mid 1980s^9–12^. Analogous to the αβ TCR, TCRγ and TCRδ chains undergo somatic recombination of variable (V), diversity (D) and joining (J) gene segments, yielding vast sequence diversity^13–15^. However, the human Vγ and Vδ repertoires are considerably smaller than those of TCRα and TCRβ. The TCRγ locus contains six functional V gene segments (Vγ2, 3, 4, 5, 8 and 9), while the TCRδ locus consists of only three bona fide V gene segments (Vδ1, 2 and 3). Other Vδ segments (such as Vδ5) arise from specific Vα genes that are infrequently used in δ-chain rearrangement^2,16^.

In addition to the differences in V gene repertoires, γδ TCRs and αβ TCRs diverge in antigen recognition. Whereas αβ TCRs mainly recognize peptide antigens presented by major histocompatibility complex (MHC) molecules, γδ TCRs can identify a diverse range of antigens in an MHC-independent manner^5,6,17–22^. For example, Vγ9Vδ2 TCRs respond to small phosphorylated non-peptide antigens, such as hydroxymethylbut-2-enyl pyrophosphate and isopentenyl pyrophosphate, produced by cellular pathogens and cancers, respectively^5^. The recognition of these phosphoantigens by Vγ9Vδ2 TCRs depends on butyrophilin 2A1 (BTN2A1) and BTN3A1 (refs. ^23–26^). Some specific Vγ5Vδ1 TCRs, such as the 9C2 γδ TCR, can recognize glycolipid antigens presented by CD1d molecules^6^.

In humans, the TCRγ and TCRδ chains associate with three CD3 dimeric subunits—CD3εγ, CD3εδ and CD3ζζ—forming an octameric γδ TCR–CD3 complex^27,28^. Each TCRγ or TCRδ contains an extracellular domain (ECD) with a Vγ/Cγ or Vδ/Cδ domain, a membrane-proximal connecting peptide (CP), a transmembrane (TM) helix and a short cytoplasmic tail. Similarly, CD3γ, CD3δ and CD3ε each encompass an ECD, a CP, a TM helix and an intracellular immunoreceptor tyrosine-based activation motif (ITAM). By contrast, CD3ζ lacks the ECD and possesses a single TM helix and three intracellular ITAMs^28^. After antigen engagement, the γδ TCR–CD3 complex triggers the phosphorylation of ITAMs within the cytoplasmic tails of CD3 subunits, initiating downstream events^2,3,29^. The γδ-TCR–CD3-mediated effector functions, along with its distinctive properties, make it an appealing target for cancer immunotherapy^4^.

Despite some existing structures of the γδ TCR ECD or complexes with their ligands^6,17,20,21,30–34^, the complete structure of the γδ TCR–CD3 complex remains unclear. Here we present two cryo-electron microscopy (cryo-EM) structures of unliganded human Vγ9Vδ2 and Vγ5Vδ1 TCR–CD3 complexes, revealing two distinct assembly mechanisms that depend on specific Vγ subset use. This assembly notably diverges from that of the conventional αβ TCR–CD3 complex, therefore offering important insights into the unique properties of the γδ TCR.

## Structure determination

**Extended Data Fig. 1:**
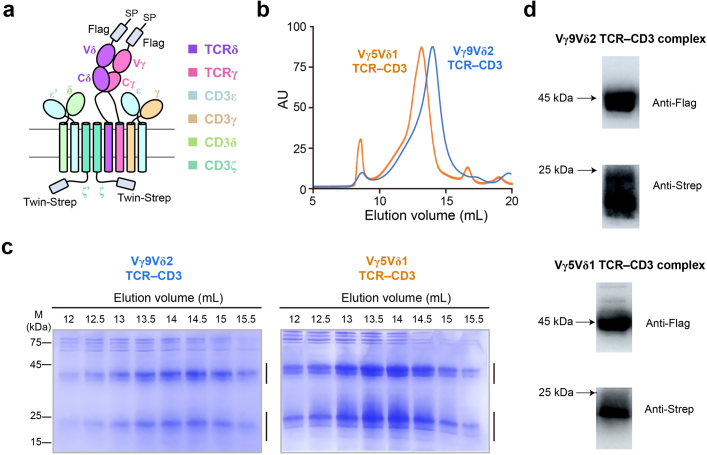
Biochemical validation of human Vγ9Vδ2 and Vγ5Vδ1 TCR–CD3 complexes. **a**, A schematic diagram of the constructs used to produce recombinant γδ TCR–CD3 complexes. SP: signal peptide. **b**, Purification of recombinant Vγ9Vδ2 and Vγ5Vδ1 TCR–CD3 complexes. The SEC chromatograms for Vγ9Vδ2 (blue) and Vγ5Vδ1 (orange) TCR–CD3 complexes are shown. AU: arbitrary units. **c**, Peak fractions were visualized on an SDS-PAGE gel stained using Coomassie blue. The bands corresponding to TCRγ9/TCRδ2 or TCRγ5/TCRδ1 (upper) and CD3ε/CD3γ/CD3δ/CD3ζ (lower) are indicated by black lines. M: protein marker. **d**, Representative results of western blots. SEC peak fractions were blotted using mouse monoclonal antibodies against Flag or Strep tags. Uncropped images in panels **c** and **d** are obtained from Supplementary Figs. 7 and 8, respectively. Results are representative of three independent experiments.

To gain a mechanistic understanding of the γδ TCR–CD3 complex assembly, we selected two prototypical human γδ TCRs—Vγ9Vδ2 and Vγ5Vδ1, derived from G115 and 9C2 clonotypes, respectively^6,31^. For simplicity, the G115 Vγ9Vδ2 and 9C2 Vγ5Vδ1 TCRs are referred to as Vγ9Vδ2 and Vγ5Vδ1 TCRs, respectively. To facilitate protein purification, we fused TCRγ and TCRδ chains and the CD3ζ subunit to affinity tags (Extended Data Fig. 1a). After affinity purification, both Vγ9Vδ2 and Vγ5Vδ1 TCR–CD3 complexes exhibited good behaviour on size-exclusion chromatography (SEC). Notably, the Vγ5Vδ1 TCR–CD3 showed a smaller elution volume compared with that of Vγ9Vδ2 TCR–CD3, implying that the Vγ5Vδ1 TCR–CD3 complex has a larger molecular mass (Extended Data Fig. 1b–d).

To evaluate the ability of our γδ TCRs in activating T cells after antigen recognition, we established a cell-based assay. We generated two Jurkat-76 cell lines^35^ that stably expressed Vγ9Vδ2 and Vγ5Vδ1 TCRs, respectively. These cells were co-cultured with K562 cells expressing BTN2A1 and BTN3A1 or CD1d as the antigen-presenting cells (APCs) (Supplementary Fig. 1a). Flow cytometry analysis revealed a substantial upregulation of CD69 in Jurkat cells expressing Vγ9Vδ2 and Vγ5Vδ1 TCRs when they were co-cultured with BTN2A1^+^BTN3A1^+^ and CD1d^+^ K562 cells, respectively, as previously reported^6,25^ (Supplementary Fig. 1b,c).

**Fig. 1:**
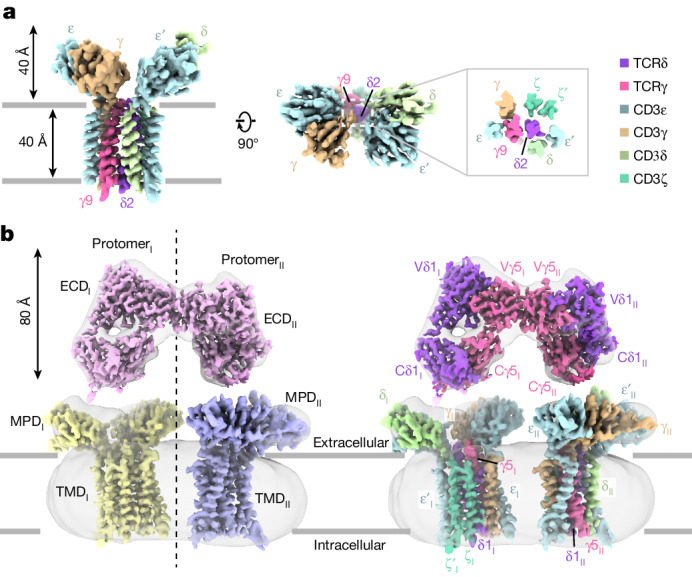
Cryo-EM reconstructions of the human Vγ9Vδ2 and Vγ5Vδ1 TCR–CD3 complexes. **a**, The structurally resolved Vγ9Vδ2 TCR–CD3 complex contains TCRγ9 (hot pink), TCRδ2 (purple), CD3ε (pale blue), CD3γ (brown), CD3δ (yellow green) and CD3ζ (teal). An extracellular view (contoured at 5*σ*) is shown. Inset: cross-section of the TMD of the Vγ9Vδ2 TCR–CD3 complex. The colour scheme in **a** is used throughout the other figures. **b**, The overall cryo-EM map (contoured at 2*σ*) of the dimeric human Vγ5Vδ1 TCR–CD3 complex. Each protomer contains an ECD, MPD and TMD. The cryo-EM maps of ECDs, MPD_I_–TMD_I_ and MPD_II_–TMD_II_, which are contoured at 9*σ*, 8*σ* and 8*σ*, respectively, are individually docked into the cryo-EM density (left). The composite cryo-EM maps were rendered using the same colour scheme as in **a** (right). All of the structural figures were prepared using UCSF ChimeraX^51^ unless otherwise indicated.

**Extended Data Fig. 2:**
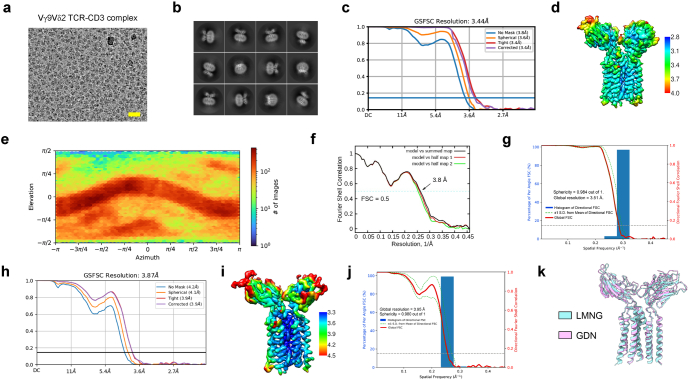
Cryo-EM analysis of the human Vγ9Vδ2 TCR–CD3 complex in LMNG or GDN detergent micelles. **a**-**b**, Representative cryo-EM micrographs (**a**) and 2D class averages (**b**) of the Vγ9Vδ2 TCR–CD3 complexes in the LMNG detergent micelles. Scale bar: 50 nm. **c**-**g**, 3D reconstruction analysis of Vγ9Vδ2 TCR–CD3 complex in the LMNG detergent micelles. The final 3D reconstruction evaluated by the 0.143 FSC criterion (blue line) (**c**). Local-resolution estimation of final 3D reconstruction sharpened by DeepEMhancer^64^. The map is contoured at 3σ (**d**). Angular distribution of the Vγ9Vδ2 TCR–CD3 complex particles in the final round of 3D refinement using CryoSPARC^59^ (**e**). FSC curves were generated to compare the refined models with the overall maps for refinement (black), as well as between each model refined in one of the two independent maps used for the FSC and the same map (red), and between the same model and the second independent map (green) (**f**). The 3DFSC sphericity plot generated using 3DFSC^66^ (**g**). **h**-**j**, 3D reconstruction analysis of Vγ9Vδ2 TCR–CD3 complex in the GDN detergent micelles. The final 3D reconstruction evaluated by the 0.143 FSC criterion (**h**). Local-resolution estimation of final 3D reconstruction. The map is contoured at 4σ (**i**). The 3DFSC sphericity plot. This plot was generated using 3DFSC^66^ (**j**). **k**, The conformations of Vγ9Vδ2 TCR–CD3 complex resolved in LMNG and GDN detergent micelles are almost the same, with a RMSD of 1.3 Å over 107 aligned Cα atom pairs.

**Extended Data Fig. 3:**
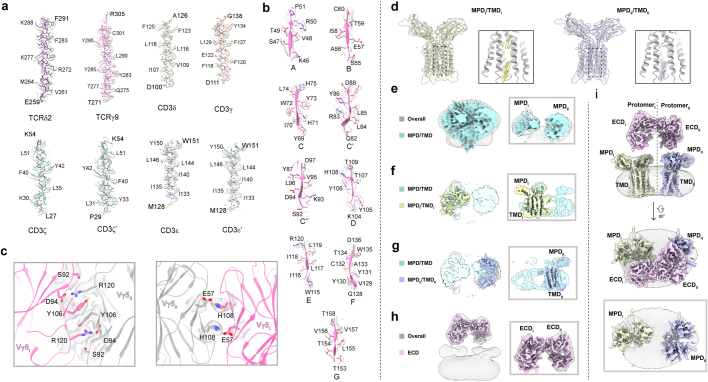
Cryo-EM density maps and model docking of human Vγ9Vδ2 or Vγ5Vδ1 TCR–CD3 complex. **a**, The representative cryo-EM densities for TMs of the Vγ9Vδ2 TCR–CD3 complex shown as transparent surface and contoured at 3σ. **b**, The cryo-EM densities for segments in one ECD of Vγ5Vδ1 TCR–CD3 complex shown as transparent surface and contoured at 3σ. **c**, Local cryo-EM density of representative interchain interface between Vγ5_I_ and Vγ5_II_ shown as transparent surface and contoured at 3σ. The model for Vγ5_I_ and Vγ5_II_ are coloured in hot pink and grey, respectively. **d**, Docking of the atomic model of Vγ9Vδ2 TCR–CD3 complex into the MPD_I_/TMD_I_ and the MPD_II_/TMD_II_ maps, respectively. Insets: the cholesterol molecule fits well with the corresponding density. **e**, Docking of MPD/TMD map into the overall map. The inset shows a good match between the features of MPD_I_ and MPD_II_ in the MPD/TMD map and those in the overall map. **f**-**g**, Docking of MPD_I_/TMD_I_ and MPD_II_/TMD_II_ maps into the MPD/TMD EM map, respectively. The inset shows a good match between the features in the TMD_I_ and TMD_II_ maps and those in the MPD/TMD map. **h**, Docking of ECD map into the overall map. The inset shows a good match between the features of the ECD map and those in the overall map. **i**, Docking of ECD, MPD_I_/TMD_I_ and MPD_II_/TMD_II_ maps into the overall map. The overall, MPD/TMD, MPD_I_/TMD_I_, MPD_II_/TMD_II_, and ECD maps are contoured at 1σ, 2σ, 8σ, 8σ, and 9σ, respectively. The details of the docking strategy are described in the method section.

After confirming the receptor activity, we conducted cryo-EM analysis of Vγ9Vδ2 and Vγ5Vδ1 TCR–CD3 complexes. For the Vγ9Vδ2 TCR–CD3 complex, we performed multiple rounds of two-dimensional (2D) and three-dimensional (3D) classification, resulting in a final 3D reconstruction at a resolution of 3.4 Å (Extended Data Fig. 2, Extended Data Table 1 and Supplementary Fig. 2). The cryo-EM map revealed well-resolved TM and membrane-proximal segments, enabling us to assign most side chains in this region (Fig. 1a and Extended Data Fig. 3a).

**Extended Data Fig. 4:**
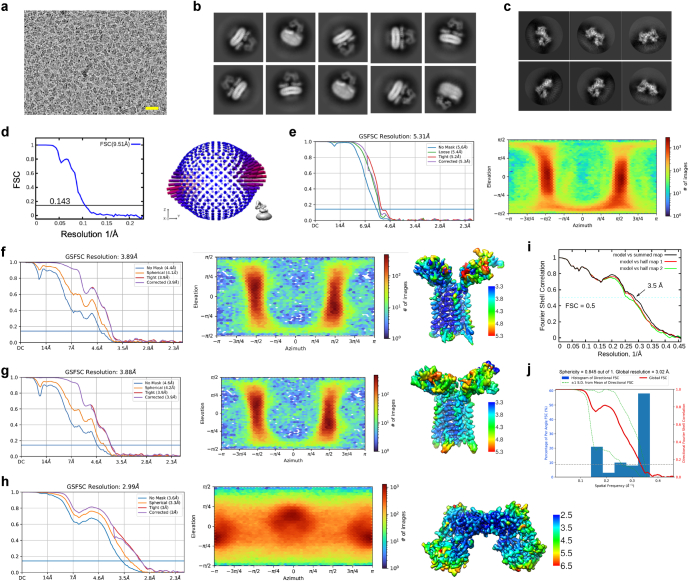
Cryo-EM analysis of the human Vγ5Vδ1 TCR–CD3 complex. **a**-**c**, Representative cryo-EM micrograph (**a**) and 2D class averages of whole particles (**b**) or MPD/TMD-subtracted ECD-recentered particles (**c**) of the human Vγ5Vδ1 TCR–CD3 complex. Scale bar: 50 nm. **d**, Cryo-EM analysis for the overall 3D reconstruction. Left: The overall 3D reconstruction shows an average resolution of 9.5 Å determined by the 0.143 FSC criterion. Right: Angular distribution of the 3D reconstruction. Each column represents one view, and the size of the column is proportional to the number of particles in that view. **e**, Cryo-EM analysis for the MPD/TMD 3D reconstruction. Left: FSC curves show an average resolution of 5.3 Å. Right: Angular distribution of the MPD/TMD particles in the final round of 3D refinement. **f**, Cryo-EM analysis for the MPD_I_/TMD_I_ 3D reconstruction. Left: FSC curves show an average resolution of 3.9 Å. Middle: Angular distribution of the MPD_I_/TMD_I_ particles in the final round of 3D refinement. Right: local resolution of the 3D reconstruction sharpened by DeepEMhancer^64^. The map is shown at 2σ. **g**, is the same as **f** but for the MPD_II_/TMD_II_ 3D reconstruction. The map is shown at 3σ. **h**, is the same as **f** but for the ECD 3D reconstruction. The map is shown at 3σ. **i**, Model versus map comparison for the ECD 3D reconstruction. FSC curves of the refined models are compared with the overall refinement maps (black), as well as to compare each model refined in one of the two independent maps used for the FSC with the same map (red), and with the second independent map (green). The marginal discrepancy between the red and green curves suggests that the model refinement process was not significantly affected by overfitting. **j**, The 3DFSC sphericity plot for the ECD 3D reconstruction. This plot was generated using 3DFSC^66^.

For the Vγ5Vδ1 TCR–CD3 complex, we used a distinct strategy. Initially, we obtained a nominal 9.5-Å-resolution cryo-EM reconstruction after several cycles of classification and refinement, which enabled us to identify two protomers, denoted I and II. Each protomer consists of an ECD, a membrane-proximal domain (MPD) and a TM domain (TMD) (Fig. 1b (left)). To achieve a higher resolution, we performed focused refinements for the ECD, MPD–TMD, as well as the individual MPD_I_–TMD_I_ and MPD_II_–TMD_II_. This yielded four additional 3D reconstructions at resolutions of 3.0 Å, 5.3 Å, 3.9 Å and 3.9 Å, respectively (Extended Data Fig. 4, Extended Data Table 2 and Supplementary Fig. 3). The high-quality cryo-EM map for the ECD enabled us to build an atomic model (Extended Data Fig. 3b,c). Although the cryo-EM densities for the MPD_I_–TMD_I_ and MPD_II_–TMD_II_ were less well resolved, we were able to dock the Vγ9Vδ2 TCR–CD3 structure into the cryo-EM densities based on clear secondary structural elements (Extended Data Fig. 3d). Ultimately, according to the features of the MPD–TMD cryo-EM maps, we docked MPD_I_–TMD_I_ and MPD_II_–TMD_II_ and their ECDs into the overall cryo-EM map, resulting in a composite map that revealed their relative arrangement (Fig. 1b (right) and Extended Data Fig. 3e–i).

## The structure of the Vγ9Vδ2 TCR–CD3 complex

**Extended Data Fig. 5:**
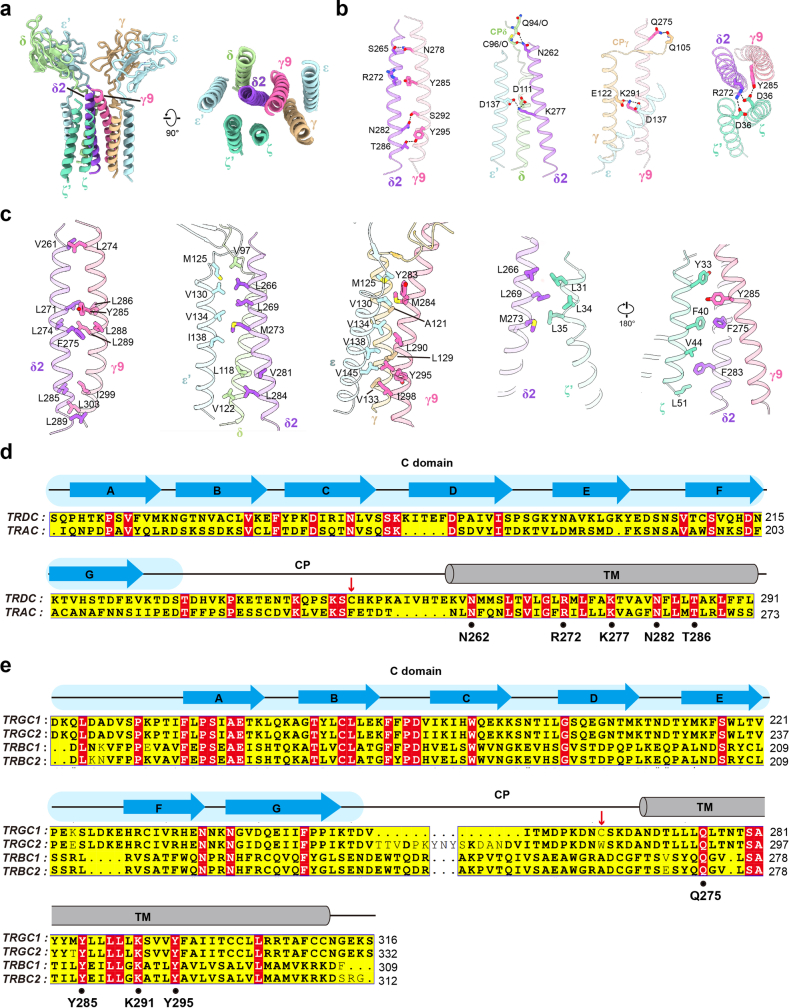
The interchain interaction of the Vγ9Vδ2 TCR–CD3 complex and the comparison of the constant regions between γδ and αβ TCRs. **a**, Overall structure of the Vγ9Vδ2 TCR–CD3 complex shown in side view (left) and top view (right) with extracellular domains hidden for clarity. **b**, The specific hydrogen bond interactions for TMs of Vγ9Vδ2 TCR–CD3 complex. The pairwise interfaces for TMs of TCRγ9/TCRδ2, TCRδ2/CD3ε’δ, TCRγ9/CD3εγ and TCRδ2γ9/CD3ζζ’ are sequentially shown from left to right panels. The hydrogen bonds are indicated by dotted lines. **c**, The extensive van der Waals interaction for the TMs of the Vγ9Vδ2 TCR–CD3 complex. The pairwise interfaces for TMs of TCRδ2/TCRγ9, TCRδ2/CD3ε’δ, TCRγ9/CD3εγ, and TCRδ2γ9/CD3ζζ’ are sequentially displayed from left to right panels. **d**, The sequence alignment of the constant regions between human TCRδ (UniProt ID: B7Z8K6) and human TCRα (UniProt ID: P01848). **e**, The sequence alignment of the constant regions between human TCRγ (UniProt ID: P03986, P0CF51) and human TCRβ (UniProt ID: P01850, A0A5B9). The residues involved in interchain interactions are indicated by black dots. The residues responsible for disulfide bond formation in Cγ1 are indicated by red arrows.

The overall structure of the Vγ9Vδ2 TCR–CD3 complex resembles a bifurcated tree, with CD3εγ and CD3ε′δ ECDs representing two primary branches and the TMD serving as the stem (Extended Data Fig. 5a). The TCRγ9 and TCRδ2 associates with CD3 subunits through their TM helices (Extended Data Fig. 5b,c). In particular, numerous membrane-embedded, interchain hydrogen bonds are observed. The interfaces of TCRγ9–TCRδ2, TCRδ2–CD3ε′δ, TCRγ9–CD3εγ and TCRγ9δ2–CD3ζζ′ register at least 13 hydrogen bonds (Extended Data Fig. 5b). Notably, these hydrogen-bond-forming residues are invariant between γδ and αβ TCRs (Extended Data Fig. 5d,e), indicating that the TMDs of αβ and γδ TCR–CD3 complexes adopt similar assembly principles.

## γδ TCR ECD and CP are greatly flexible

**Fig. 2:**
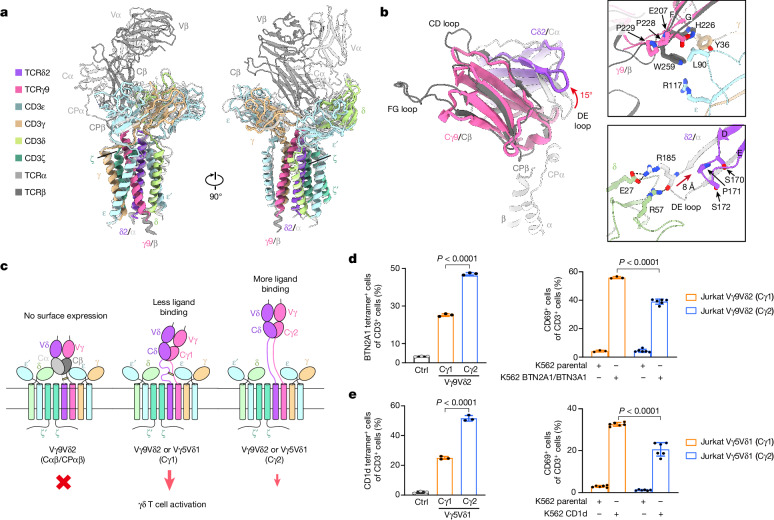
The γδ TCR ECD lacks the interaction with CD3 ECDs in Vγ9Vδ2 TCR–CD3. **a**, Structural comparison between the Vγ9Vδ2 and αβ TCR–CD3 complex (Protein Data Bank (PDB): 7FJD)^40^. Two perpendicular views are shown. **b**, Structural comparison between the Cα/Cβ and Cδ/Cγ domains (PDB: 1HXM)^31^. Insets: magnified views of the interface of TCRγ9–CD3εγ or TCRβ–CD3εγ ECDs (top right), and the interface of TCRδ2–CD3δ or TCRα–CD3δ ECDs (bottom right). Hydrogen bonds are indicated by black dashed lines. The conformational changes of the DE loop of the Cδ2 domain are indicated by red arrows. **c**, Three TCR variants were investigated: Vγ9Vδ2 TCR(Cαβ/CPαβ), Vγ9Vδ2 TCR(Cγ1) and Vγ9Vδ2 TCR(Cγ2). These variants exhibited diverse ligand-association abilities and distinct intensities of TCR signalling. **d**, Quantification of human BTN2A1 tetramer staining (left; *n* = 3 per group) and T cell activation after 24 h of co-culture with K562 cells expressing BTN2A1 and BTN3A1 or ZIM3–dCas9 (ref. ^52^) (parental) (right; *n* = 3 per Cγ1 group; *n* = 6 per Cγ2 group). Jurkat cells expressing Vγ5Vδ1 TCR were used as the negative control (Ctrl). **e**, Quantification of human CD1d–α-GalCer tetramer staining (left; *n* = 3 per group) and T cell activation after 24 h of co-culture with K562 cells expressing CD1d or ZIM3–dCas9 (ref. ^52^) (parental) (right; *n* = 6 per group). Jurkat cells expressing Vγ9Vδ2 TCR were used as the negative control. In **d** and **e**, results are representative of three independent experiments and each symbol corresponds to a biologically independent experiment. Data are mean ± s.d. *P* values were calculated using one-way analysis of variance (ANOVA) followed by Dunnett’s multiple-comparison test. Bar graphs throughout the Article were created using GraphPad Prism 9. Source Data

The resolved portion of the Vγ9Vδ2 TCR–CD3 structure approximately resembles the αβ TCR–CD3 structure, with a root mean squared deviation of 0.8 Å over 240 aligned Cα atoms (Fig. 2a). As predicted previously^1^, the TCRδ2 and TCRγ9 TM helices occupy positions corresponding to TCRα and TCRβ, respectively. However, outside this region, there are substantial differences. In the αβ TCR–CD3 complex, the TCRα and TCRβ ECDs stack against CD3 ECDs through Cα and Cβ domains, and their CPs tightly bind to the Cα and Cβ domains. By contrast, the ECDs and CPs of TCRγ9 and TCRδ2 are entirely invisible, most likely due to the lack of interaction between the Cδ and Cγ domains and CD3 subunits (Fig. 2a).

To elucidate why Cδ and Cγ domains do not interact with CD3 ECDs, we superimposed the crystal structure of Cδ and Cγ domains onto the αβ TCR–CD3 structure and analysed the interfaces (Fig. 2b). We observed that Cδ2 domains display a noticeable distinction in the DE loop (Fig. 2b (left)). A closer examination revealed that hydrogen bonds formed by residues between TCRα and CD3δ may be eliminated due to an 8 Å backward shift of DE loop (Fig. 2b (bottom right)). A similar situation occurs at the Cβ–CD3εγ ECD interface. His226 and Trp259 in TCRβ are replaced by Pro228, Pro229 and Glu207 in TCRγ9, which may disrupt the interactions between the Cγ domain and CD3εγ ECDs (Fig. 2b (top right)). Thus, the primary sequence and conformation differences between the Cα/Cβ and Cδ/Cγ domains probably result in the dissociation of the TCRγ and TCRδ ECD from CD3 ECDs.

## The regulatory roles of CP

To examine the impact of the flexibility of the γδ TCR ECD, we designed a chimera TCR by replacing the CP and C domains with those from the αβ TCR (Fig. 2c). We anticipated that the chimera ECDs (Vγ9Vδ2–Cα/Cβ) would interact with the CD3 ECDs, thereby restricting the mobility of the ECD. Moreover, to enhance TCR ECD flexibility, we substituted the original TCRγ(Cγ1) with the other isoform, TCRγ(Cγ2). In contrast to αβ TCR, the two isoforms exhibit apparent sequence differences in CP (Extended Data Fig. 5e). Cγ2 contains an additional 16 amino acids in the CP and does not form a disulfide bond with Cδ^36,37^. The longer, non-disulfide-linked CP is expected to confer greater conformational flexibility to the Vγ9Vδ2 TCR ECD (Fig. 2c).

**Extended Data Fig. 6:**
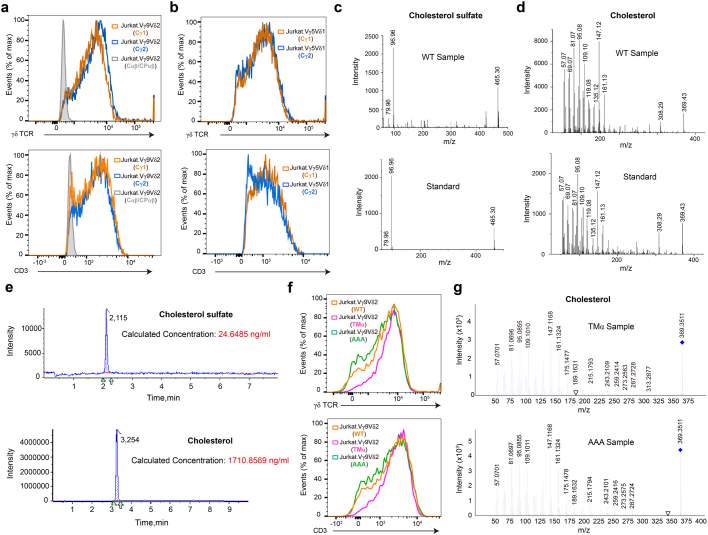
Cellular and biochemical characterizations of wildtype and mutant γδ TCR–CD3 complexes. **a**, The expression level of γδ TCR or CD3 on Jurkat-76 cells expressing Vγ9Vδ2 TCR (Cαβ/CPαβ), Vγ9Vδ2 TCR (Cγ1), and Vγ9Vδ2 TCR (Cγ2). **b**, The expression level of γδ TCR or CD3 on Jurkat-76 cells expressing Vγ5Vδ1 TCR (Cγ1), and Vγ5Vδ1 TCR (Cγ2). **c**, Quantitative detection of cholesterol sulfate by LC–MS/MS. The characteristic peaks of cholesterol sulfate in the Vγ9Vδ2 TCR–CD3 complex sample and the standard sample, denoted by numerals. Specifically, the peak at m/z 465.30 corresponding to parent ion [M + H-H2O]^+^. **d**, Quantitative detection of cholesterol by LC–MS/MS. The characteristic peaks of cholesterol in the Vγ9Vδ2 TCR–CD3 complex sample and the standard sample, denoted by numerals. Specifically, the peak at m/z 369.43 corresponding to parent ion [M + H-H2O]^+^. **e**, The representative chromatogram of cholesterol sulfate (up) and cholesterol (down) extracted from a purified Vγ9Vδ2 TCR–CD3 complex sample. The abundance of cholesterol is nearly 70-fold higher than that of cholesterol sulfate in purified Vγ9Vδ2 TCR–CD3 proteins. **f**, The expression level of γδ TCR or CD3 on Jurkat-76 cells expressing Vγ9Vδ2 TCR (WT), Vγ9Vδ2 TCR (TMα), and Vγ9Vδ2 TCR (AAA). Numbers in plots indicate percent of gated events. **g**, The characteristic peaks of cholesterol in the Vγ9Vδ2 (TMα) TCR–CD3 and Vγ9Vδ2 (AAA) TCR–CD3 complex sample. Data are representative of two independent experiments.

We next compared the functional distinctions of the three TCR variants: Vγ9Vδ2 TCR(Cαβ/CPαβ), Vγ9Vδ2 TCR(Cγ1) and Vγ9Vδ2 TCR(Cγ2). We focused on three key aspects: (1) TCR expression level; (2) ligand tetramer binding ability; and (3) CD69 upregulation after co-culture with APCs. First, Vγ9Vδ2-TCR(Cαβ/CPαβ)^+^ Jurkat cells showed no detectable TCR expression, whereas Vγ9Vδ2-TCR(Cγ1)^+^ and Vγ9Vδ2-TCR(Cγ2)^+^ Jurkat cells displayed adequate and comparable levels of TCR expression (Extended Data Fig. 6a). This suggests that the CPs and Cδ/Cγ have a critical role in γδ TCR surface expression. Next, Vγ9Vδ2-TCR(Cγ2)^+^ cells exhibited higher avidity to BTN2A1 tetramers (Fig. 2d). This can be attributed to the increased flexibility of the Cγ2, which facilitates easier access of the TCR ECD to BTN2A1 tetramers. Notably, despite the enhanced tetramer binding, Vγ9Vδ2 TCR(Cγ2)^+^ cells displayed lower CD69 upregulation after co-culture with APCs (Fig. 2d). This suggests that excessive ECD flexibility (or the longer CP) may impede T cell activation. Notably, similar functional differences between Cγ1 and Cγ2 were observed in cells expressing Vγ5Vδ1 TCR (Fig. 2e and Extended Data Fig. 6b).

## The inhibitory effects of cholesterol

**Fig. 3:**
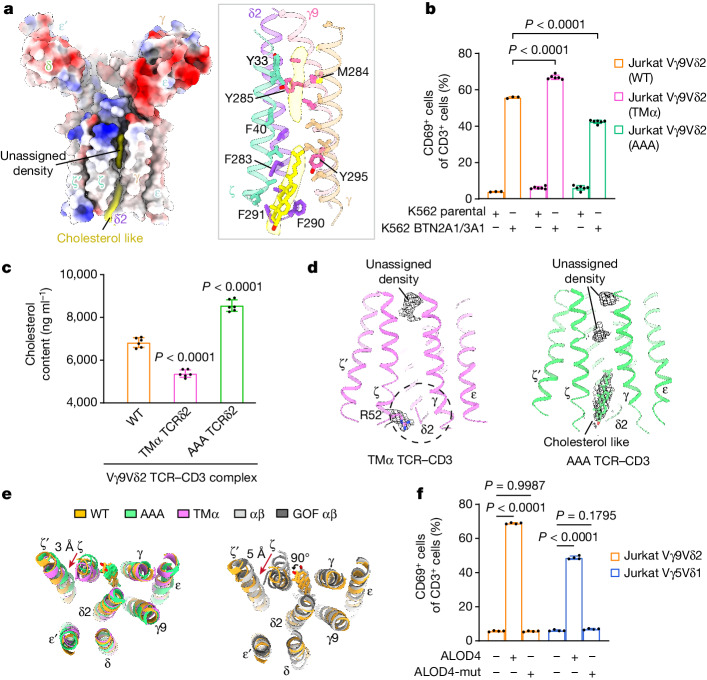
The cholesterol molecule has an inhibitory role in TCR signalling. **a**, Two molecules (cholesterol-like and unassigned densities) were embedded into the TMD of the Vγ9Vδ2 TCR–CD3 complex. The electrostatic surface potential map of the Vγ9Vδ2 TCR–CD3 complex (left) and a magnified view of the interactions between the cholesterol-like molecules and the complex (right) are shown. The cryo-EM densities are contoured at 9*σ*. **b**, Flow cytometry analysis of CD69 expression on Jurkat-76 cells transduced with WT (*n* = 3 per group) and mutant variants of Vγ9Vδ2 TCR (*n* = 6 per group) after co-culture for 15 h with K562 cells expressing CD1d or ZIM3–dCas9 (ref. ^52^) (parental). **c**, Quantitative analysis of cholesterol content in purified WT or mutant Vγ9Vδ2 TCR–CD3 complex using liquid chromatography coupled with tandem MS (LC–MS/MS; *n* = 6 per group). **d**, Magnified views of the TMD of the TMα and AAA Vγ9Vδ2 TCR–CD3 complex. The cryo-EM maps are shown as a black mesh and contoured at 8*σ*. The position of the cholesterol binding site in the Vγ9Vδ2 TCR–CD3 complex is indicated by a dashed circle. **e**, Structural comparison of the TMDs of the WT, AAA and TMα Vγ9Vδ2 TCR–CD3 complex (left). Right, structural comparison of the TMDs of Vγ9Vδ2, WT αβ (PDB: 7FJD)^40^ and gain-of-function (GOF) αβ TCR–CD3 complexes (PDB: 7FJE)^40^. **f**, Flow cytometry analysis of CD69 expression on Jurkat-76 cells that were transduced with Vγ9Vδ2 TCR and Vγ5Vδ1 TCR, with or without treatment with 0.5 μM ALOD4 and 0.5 μM ALOD4 non-binding mutant (ALOD4-mut) for 12 h. *n* = 4 per group. Results are representative of three (**b** and **f**) and two (**c**) independent experiments. Each symbol corresponds to a biologically independent experiment. Data are mean ± s.d. *P* values were calculated using one-way ANOVA with Dunnett’s multiple-comparison test. For **c**, mutant complexes were compared with the WT complex. Source Data

In the Vγ9Vδ2 TCR–CD3 TMD, we observed cholesterol-like densities that wedge into hydrophobic clefts formed by TMs of CD3ζ, CD3γ, TCRδ2 and TCRγ9 (Fig. 3a). The mass spectrometry (MS) results suggest that the abundance of cholesterol is around 70-fold higher compared with that of cholesterol sulfate (Extended Data Fig. 6c–e), both of which may bind to the TCR–CD3 complex^38–40^. The densities at the upper cleft were not consistently discernible during the data processing. Thus, we tentatively assigned cholesterol molecules into the densities at the lower cleft. These cholesterol-like molecules are buttressed by a number of bulky residues, exemplified by Phe283, Phe290 and Phe291 in TCRδ2 and Tyr295 in TCRγ9 at the lower cleft (Fig. 3a). The cholesterol-like densities were also observed in similar positions in the Vγ5Vδ1 TCR–CD3 complex (Extended Data Fig. 3d).

To investigate the role of the cholesterol-like molecule, we introduced a number of structure-guided mutations into TCRγ9 or TCRδ2 and evaluated their functional consequences using our cell-based assay. Most mutants exhibited notable alterations in TCR expression levels, making them unsuitable for assessing the functional effects. However, we successfully identified two strains of Jurkat cells—one carrying F283A, F290A and F291A in TCRδ2 (AAA); and the other carrying the TM of TCRα (Ile254–Ser273) replacing the TM (Met273–Leu292) in TCRδ2 (TMα). These strains exhibited comparable levels of TCR expression to those of WT Vγ9Vδ2 Jurkat cells (Extended Data Fig. 6f). After stimulation by APCs, the Jurkat cells expressing the AAA and TMα TCRs displayed significantly lower and higher CD69 expression, respectively, compared with those expressing wild-type (WT) TCRs (Fig. 3b).

**Extended Data Fig. 7:**
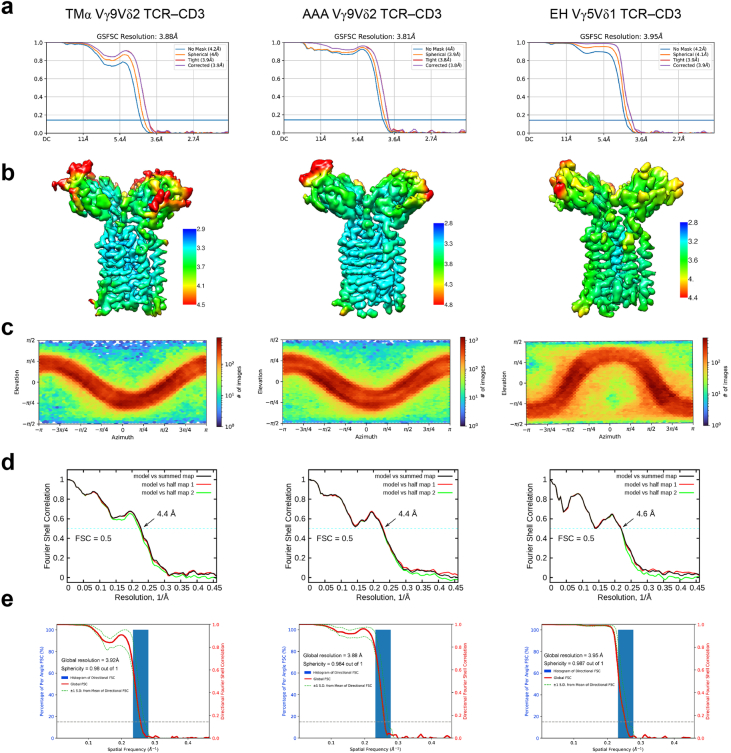
Analysis of the 3D reconstructions of the mutant Vγ9Vδ2 and mutant Vγ5Vδ1 TCR–CD3 complexes. **a**-**b**, Gold standard FSC curves (**a**) and local resolution estimation (**b**) of 3D reconstructions of TMα mutant Vγ9Vδ2 TCR–CD3 complex (left), AAA mutant Vγ9Vδ2 TCR–CD3 complex (middle), and EH mutant Vγ5Vδ1 TCR–CD3 complex (right). Maps were sharpened by DeepEMhancer. **c**, Angular distribution of the TMα mutant Vγ9Vδ2 TCR–CD3 complex (left), AAA mutant Vγ9Vδ2 TCR–CD3 complex (middle), and EH mutant Vγ5Vδ1 TCR–CD3 complex (right) complex particles in the final round of 3D refinement using CryoSPARC. **d**, Model versus map comparison for the final 3D reconstruction of the TMα mutant Vγ9Vδ2 TCR–CD3 complex (left), AAA mutant Vγ9Vδ2 TCR–CD3 complex (middle), and EH mutant Vγ5Vδ1 TCR–CD3 complex (right). FSC curves of the refined models are compared with the overall refinement maps (black), as well as to compare each model refined in one of the two independent maps used for the FSC with the same map (red), and with the second independent map (green). The marginal discrepancy between the red and green curves suggests that the model refinement process was not significantly affected by overfitting. **e**, The 3DFSC sphericity plot for the final 3D reconstruction of the TMα mutant Vγ9Vδ2 TCR–CD3 complex (left), AAA mutant Vγ9Vδ2 TCR–CD3 complex (middle), and EH mutant Vγ5Vδ1 TCR–CD3 complex (right). This plot was generated using 3DFSC.

To assess the impact of these mutations on cholesterol binding to Vγ9Vδ2 TCR–CD3, we conducted a quantitative MS analysis (Fig. 3c and Extended Data Fig. 6g). Compared with the WT complex, the AAA complex displayed elevated cholesterol levels, whereas the TMα complex exhibited reduced levels. Cryo-EM structures of the two mutant complexes are consistent with this result; the cholesterol-like density in the AAA complex is preserved within the cleft, whereas the density in the TMα complex is diminished due to the steric hindrance caused by Arg52 in CD3ζ and the C terminus of TCRδ2 (Fig. 3d, Extended Data Fig. 7, Supplementary Fig. 4a,b and Extended Data Table 3). Considering the functional properties of these two mutants in T cell activation (Fig. 3b), we propose that cholesterol bound to the Vγ9Vδ2 TCR–CD3 complex exerts an inhibitory effect on TCR signalling.

By comparing the structures of three Vγ9Vδ2 TCR–CD3 variants, we observed distinct conformations in the TM helix of CD3ζ. Concurrent with the absence of cholesterol, the proximal C terminus of the CD3ζ TMD undergoes a lateral shift of 3 Å from AAA to TMα complexes (Fig. 3e). Notably, the conformations of CD3ζ in the AAA and TMα complexes resemble those in the WT αβ and the gain-of-function αβ TCR–CD3 (ref. ^40^), respectively (Fig. 3e). This structural observation is consistent with previous Förster resonance energy transfer (FRET) data showing that TCR engagement leads to the rearrangement of the CD3ζ/ζ′ juxtamembrane regions^41^.

To further substantiate the inhibitory effects of cholesterol on TCR signalling, we performed a cholesterol-depletion assay using ALOD4 treatment in Jurkat cells. ALOD4 can selectively sequester cholesterol within the plasma membrane while not affecting cellular cholesterol levels^42^. After treatment with ALOD4, we observed a substantial upregulation in CD69 expression in both Vγ9Vδ2- and Vγ5Vδ1-expressing Jurkat cells compared with those treated with ALOD4 non-binding mutant (Fig. 3f). This assay reinforces the notion that cholesterol exerts inhibitory effects on γδ TCR signalling, a similar mechanism to αβ TCR^38,40^.

## The structure of the Vγ5Vδ1 TCR–CD3 complex

**Fig. 4:**
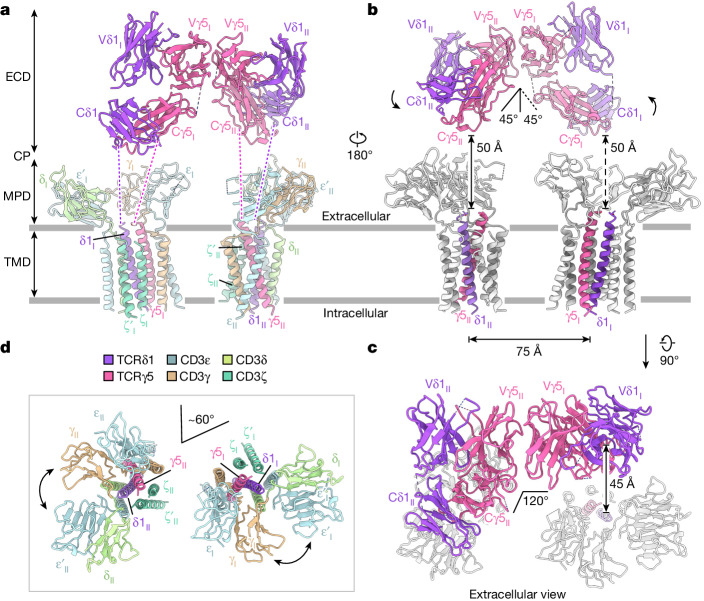
The Vγ5Vδ1 TCR–CD3 complex is an asymmetric dimer. **a**, The Vγ5Vδ1 TCR–CD3 complex displays an asymmetric dimeric assembly. Two protomers are nestled against each other through the Vγ5 domains. The unresolved loops in the Vγ5Vδ1 TCR–CD3 complex are indicated by dashed lines. **b**, TCRγ5 and TCRδ1 are asymmetrically arranged. The ECD of Vγ5Vδ1 TCR–CD3 pivots around along the axis vertical to the membrane, suspending over the membrane. The vertical distance between the ECD and TMD is indicated by solid and dashed lines. **c**, An extracellular view of the Vγ5Vδ1 TCR–CD3 complex. The Cγ5_II_ and Cδ1_II_ domains are positioned directly above the TMD_II_, while the Cγ5_I_ and Cδ1_I_ domains display a lateral deviation by about 45 Å from the TMD_I_. **d**, MPD_I_–TMD_I_ and MPD_II_–TMD_II_ are arranged with a relatively flexible angle. An extracellular view is shown.

The Vγ5Vδ1 TCR–CD3 complex displays a dimeric assembly, comprising two protomers nestled back-to-back through their ECDs, with each MPD and TMD independently embedded in the membrane. The ECD, MPD and TMD are respectively composed of Vδ/Vγ/Cδ/Cγ domains, CD3 ECDs, and TCRγ5, TCRδ1 and CD3 TMDs. In each protomer, the ECD and MPD–TMD are connected by unresolved TCRγ5 and TCRδ1 CPs (Fig. 4a). The ECD_I_ and ECD_II_, interacting through their Vγ5 domains, are oriented perpendicularly and elevated over the membrane by around 50 Å (Fig. 4b). In the overall configuration of the complex, ECD_I_–ECD_II_ pivots around the Vγ5_I_–Vγ5_II_ interface by about 45° along an axis perpendicular to the membrane. Underneath the ECDs, the two TMDs are separated by about 75 Å and appear to have no direct interactions (Fig. 4b). Notably, in protomer II, the Cγ5 and Cδ1 domains are aligned vertically with the TMD, whereas, in protomer I, the Cγ5 and Cδ1 domains laterally deviate from the TMD by about 45 Å (Fig. 4c).

MPD_I_–TMD_I_ and MPD_II_–TMD_II_ are arranged asymmetrically, with CD3ζ_II_ζ′_II_ facing CD3ε_I_ (Fig. 4d). Our current model suggests that MPD_I_–TMD_I_ and MPD_II_–TMD_II_ are positioned at an angle of around 60° relative to each other. However, the MPD–TMD region is resolved at a moderate resolution in the overall cryo-EM map, suggesting substantial conformational heterogeneity. It is possible that MPD_I_–TMD_I_ and MPD_II_–TMD_II_ are mobile rather than fixed in position. Despite this, we docked individual MPD–TMD maps into the overall map using their features (Extended Data Fig. 3e–i).

## The dimer interface

**Fig. 5:**
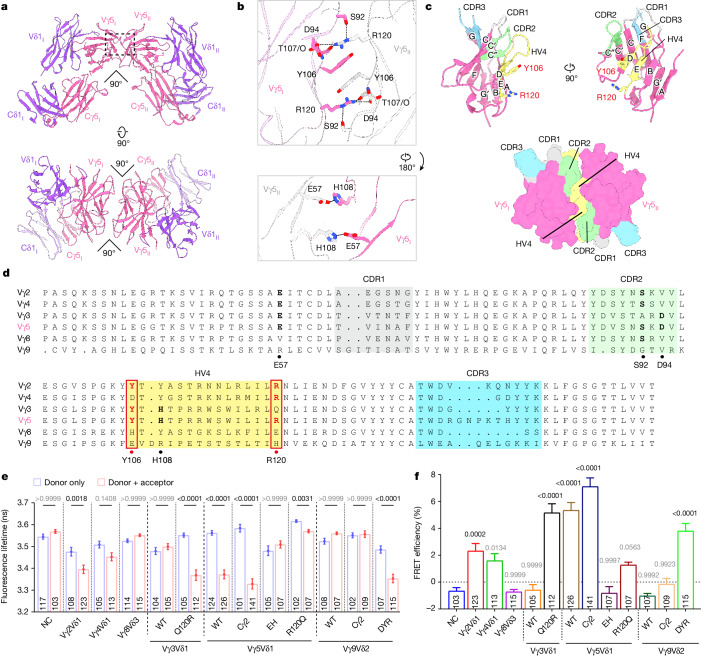
The dimerization interface of the Vγ5 domains is composed of a germline-encoded V region. **a**, The ECD in the Vγ5Vδ1 TCR–CD3 complex displays a dimer with two-fold symmetry. Two perpendicular views are shown. The dimerization interface is indicated by a dashed square and the detailed interaction is depicted in part **b**. **b**, Two magnified views are shown for the interfaces between Vγ5_I_ and Vγ5_II_. Hydrogen bonds are indicated by black dashed lines. The Vγ5_II_ domain is coloured grey for clarity. T107/O, the carbonyl oxygen of Thr107. **c**, Mapping the CDR1, CDR2, CDR3 and HV4 regions onto the dimeric Vγ5 domains. The CDR1, CDR2, CDR3 and HV4 regions are coloured grey, pale green, cyan and yellow, respectively. Top, two perpendicular views of Vγ5 domains. Bottom, the surface of the Vγ5_I_–Vγ5_II_ domains. The dimeric interface is conferred primarily by the HV4 region with some contributions from the CDR2 region. **d**, Sequence alignment among different human Vγ domains (UniProt: A0A075B6R0, A0A0C4DH28, P03979, A0A0B4J1U4, A0A0C4DH27 and Q99603). The residues involved in Vγ5_I_–Vγ5_II_ interactions are indicated in bold. The key residues that are responsible for dimerization are indicated by red font. **e**, The fluorescence lifetime of moxCerulean3 was measured in HEK293T cells using FLIM–FRET. Donor only, the fluorescence lifetime in cells that were transfected with moxCerulean3-labelled complex. Donor + acceptor, the lifetime in cells co-expressing moxCerulean3-labelled and mGold-labelled complex. **f**, The FRET efficiency was determined in cells expressing different γδ TCR–CD3 complexes. The mean of each donor only group was used to calculate the FRET efficiency in each donor + acceptor group. Results in **e** and **f** are representative of two independent experiments. *n* values representing biologically independent cells is provided for each group. Data are mean ± s.e.m. *P* values were calculated using one-way ANOVA with Kruskal–Wallis test (**e**) and by one-way ANOVA with Dunnett’s multiple-comparison test (**f**). For **f**, the FRET efficiency of each γδ TCR–CD3 complex was compared with that of the negative control (NC) group. Source Data

The ECDs in our intact complex exhibit a dimer with two-fold symmetry, with an interface consistent with that in previous crystal structure^6^ (Fig. 5a). Examination of the Vγ5_I_–Vγ5_II_ interface reveals intricate interactions, primarily involving a pair of interlocked Tyr106 and Arg120 residues (Fig. 5b). Tyr106 and Arg120 in Vγ5_I_ interact with the opposite Arg120 and Tyr106 in Vγ5_II_, resulting in cation–π and π–π interactions among the four residues (Arg-Tyr-Tyr-Arg) (Fig. 5b). Furthermore, the guanidinium group of Arg120 in one Vγ5 domain donates three hydrogen bonds to Ser92, Asp94 and Thr107 in the other Vγ5 domain. On the opposite side of the Arg-Tyr-Tyr-Arg cluster, a pair of hydrogen bonds formed by Glu57 and His108 is also observed (Fig. 5b and Supplementary Table 1).

The Vγ domain comprises four hypervariable (HV) regions, namely complementarity-determining region 1 (CDR1), CDR2, CDR3 and HV4. CDR1, CDR2 and HV4 are encoded by inherited V-gene segments, whereas CDR3 is generated by imprecise somatic gene rearrangement of the VJ region^7,13^. We found that the Vγ5_I_–Vγ5_II_ interface primarily involves HV4, with some contributions from CDR2 (Fig. 5c). Specifically, Tyr106, Arg120 and His108 are located within the HV4 region. Other interacting residues are found in CDR2 (Ser92 and Asp94) and the V-gene-conferred region (Glu57) (Fig. 5d).

To elucidate the assembly mechanisms of other human γδ TCR–CD3 complexes, we compared the protein sequences of different human Vγ domains (Fig. 5d). Tyr106 and Arg120, which are conserved in the Vγ5 domain, are also conserved in the Vγ2 domain. Conversely, the Vγ3, Vγ4, Vγ8 and Vγ9 domains exhibit variability at these positions. This indicates that the γδ TCR–CD3 complex with the Vγ5 or Vγ2 domain may form dimers, while the others may be monomeric.

To test this hypothesis, we used fluorescence lifetime imaging microscopy (FLIM)–FRET analysis to investigate the oligomerization states of γδ TCR–CD3 complexes on the cell membrane^43^. In cells expressing Vγ4Vδ1, Vγ8Vδ3, Vγ3Vδ1, Vγ9Vδ2 and Vγ9Vδ2(Cγ2) TCR–CD3 complexes, the lifetime of CFP fluorescence was similar to those expressing TCRγ–CFP alone (Fig. 5e). These data implicate that these TCR–CD3 complexes are spatially separated and probably exist as monomers. By contrast, cells expressing Vγ2Vδ1, Vγ5Vδ1 and Vγ5Vδ1(Cγ2) TCR–CD3 complexes exhibited a significant reduction in the lifetime of CFP fluorescence (Fig. 5e), implying that these TCR–CD3 complexes are in close proximity, probably existing as dimers. Nevertheless, the FRET data alone do not preclude the possibility of a mixture of monomers, dimers and higher-order oligomers for the TCR–CD3 complex on the cell membrane.

**Extended Data Fig. 8:**
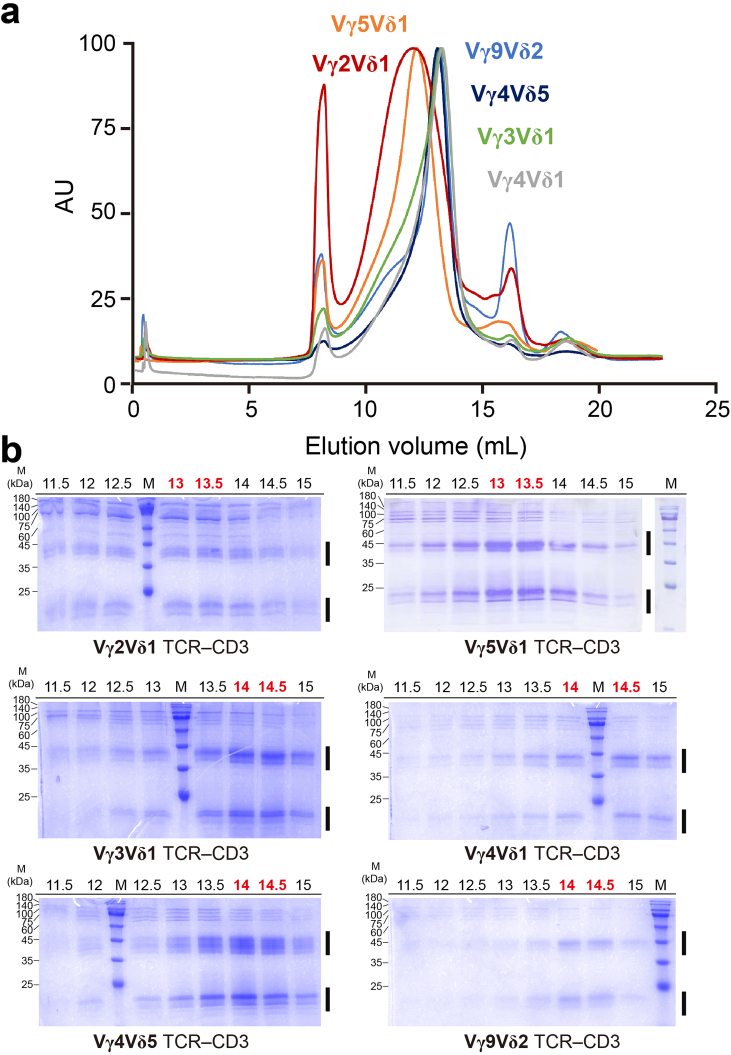
SEC analysis of human γδ TCR–CD3 complexes with different Vγ subsets. **a**, The chromatogram shows that the elution volume of Vγ2Vδ1 (red) or Vγ5Vδ1 (orange) TCR–CD3 complexes is notably smaller than that of Vγ9Vδ2 (blue), Vγ4Vδ5 (dark blue), Vγ3Vδ1 (green), or Vγ4Vδ1 (grey) TCR–CD3 complexes. **b**, Peak fractions were visualized on an SDS-PAGE gel through Coomassie staining. The elution volume of each fraction is labelled in mL on the top of each lane. The two most intense fractions containing γδ TCR–CD3 complexes are highlighted by red bold. The TCRγ/TCRδ chains (upper) and CD3 subunits (lower) are indicated by black line on the right of the gel. M: protein maker. Uncropped images are obtained from Supplementary Fig. 9. Results are representative of two independent experiments.

**Extended Data Fig. 9:**
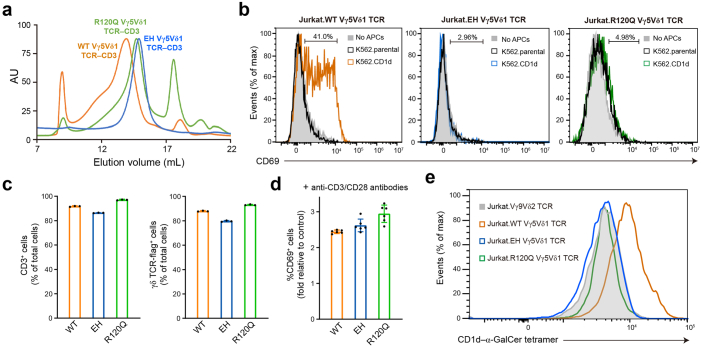
Monomeric mutant Vγ5Vδ1 TCR–CD3 complex fails to initiate T cell activation. **a**, SEC analysis of recombinant WT, R120Q and EH Vγ5Vδ1 TCR–CD3 complexes. The SEC chromatograms for WT (orange), R120Q (green), and EH (blue) Vγ5Vδ1 TCR–CD3 complexes are shown. AU: arbitrary units; WT: wild-type; R120Q: R120Q mutation in TCRγ5 chain; EH: Y106E/R120H mutations in TCRγ5 chain. **b**, Flow cytometry analysis of CD69 expression on WT (left) or EH (middle) or R120Q (right) γδ TCR-transduced Jurkat-76 cells cocultured with K562 cells expressing CD1d or ZIM3–dCas9 (ref. ^52^) (parental) or without K562 cells. Numbers in plots indicate percent of gated events. APC: antigen-presenting cells. **c**, Flow cytometry analysis of CD3 (left) or γδ TCR (right) expression level on Jurkat-76 cells expressing WT (orange), R120Q (green), or EH (blue) Vγ5Vδ1 TCRs (n = 3 per group). The surface expression level of γδ TCRs was detected by anti-Flag antibodies. **d**, Flow cytometry analysis of CD69 upregulation on WT (orange), R120Q (green), or EH (blue) Vγ5Vδ1 TCR-transduced Jurkat-76 cells cocultured with anti-CD3/CD28 antibodies in 24 h (n = 6 per group). Results are presented as the proportion of CD69^+^ cells (%CD69^+^ cells) in each experimental co-culture relative to that in the control co-culture. **e**, Flow cytometry analysis of Jurkat-76 cells expressing the γδ TCRs of interest and stained by human CD1d–α-GalCer tetramers. Numbers in plots indicate percent of gated events. Results are representative of three independent experiments in **b**–**d**, and two independent experiments in **e**. In panels **c** and **d**, each symbol represents a biologically independent experiment and data are represented as mean ± SD. Source Data

To further confirm the oligomeric state, we introduced the Y106E/R120H (EH) and R120Q mutations in the Vγ5 domain to disrupt the dimer interface (Fig. 5d). Consistent with our analysis, the FRET signal exhibited a significant reduction in cells expressing these mutant complexes (Fig. 5e,f). Furthermore, we conducted a swapping assay to engineer Vγ9Vδ2 or Vγ3Vδ1 complexes forming dimers. We generated two variants, each carrying a V94D/E106Y/H120R mutation in Vγ9 or a Q120R mutation in Vγ3. The cells expressing these mutant complexes show notable FRET signals (Fig. 5e,f). Importantly, our biochemical and structural characterizations are consistent with these cell-based FRET results (Extended Data Figs. 7–9a, Extended Data Table 3 and Supplementary Fig. 4c).

## The dimeric Vγ5Vδ1 TCR–CD3 is functional

To examine whether our dimeric Vγ5Vδ1 TCR–CD3 structure is a functional state rather than an artificial one, we assessed the function of monomeric EH or R120Q Vγ5Vδ1 TCRs in vivo. We found that EH or R120Q Vγ5Vδ1 TCR^+^ Jurkat cells did not show any augmentation in CD69 expression after co-culture with APCs (Extended Data Fig. 9b). This suggests that the EH or R120Q Vγ5Vδ1 TCR is incapable of initiating T cell activation. Thus, we demonstrate that dimeric WT Vγ5Vδ1 TCR, but not monomeric EH or R120Q mutant, is required for T cell activation.

To investigate why the monomeric mutant TCR does not initiate T cell activation, we first examined the expression levels of EH or R120Q Vγ5Vδ1 TCR and CD3 subunits on the plasma membrane. Both EH and R120Q Vγ5Vδ1 TCR^+^ Jurkat cells exhibited sufficient surface expression (Extended Data Fig. 9c). Moreover, EH and R120Q Vγ5Vδ1 TCR^+^ Jurkat cells displayed adequate upregulation of CD69 after stimulation with anti-CD3 antibodies (Extended Data Fig. 9d). Using a ligand tetramer-binding assay, we found that WT Vγ5Vδ1 TCR^+^ Jurkat cells displayed strong staining with CD1d–α-GalCer tetramers, whereas EH or R120Q Vγ5Vδ1 TCR^+^ Jurkat cells showed no detectable staining (Extended Data Fig. 9e). This indicates that EH or R120Q Vγ5Vδ1 TCRs on the native plasma membrane display very weak avidity towards CD1d–α-GalCer, thereby preventing T cell activation.

Structural analysis reveals that the WT Vγ5Vδ1 TCR–CD3 structure permits docking of two CD1d molecules without any steric hindrance (Supplementary Fig. 5a). Compared with the WT complex, which possesses two binding sites for the CD1d–α-GalCer tetramer, the EH or R120Q TCR–CD3 complex has only one binding site (Supplementary Fig. 5b). Consequently, the mutant TCR–CD3 complexes probably exhibit substantially reduced binding avidity towards the ligand tetramer. The distinction between bivalent and monovalent binding provides a plausible explanation for the lack of surface staining of the monomeric TCR mutants.

## Discussion

The structures of human G115 Vγ9Vδ2 and 9C2 Vγ5Vδ1 TCR–CD3 complexes reveal a stoichiometry of TCRγδ–CD3ε_2_γδζ_2_ (Fig. 1), confirming the previous model^27^. Our structures revealed two distinct assembly mechanisms that differ from those of αβ TCR–CD3 complexes (Figs. 2 and 3). Notably, we identified a dimeric assembly in the Vγ5Vδ1 TCR–CD3 complex, whereby dimerization depends on the Vγ5 domain (Figs. 4 and 5). Furthermore, we performed comprehensive assays and demonstrated that the dimeric form of the Vγ5Vδ1 TCR–CD3 complex is responsible for T cell activation (Extended Data Fig. 9).

The conformational flexibility of the γδ TCR ECD may enable γδ TCR to recognize ligands using distinct docking geometries. Consistent with this scenario, CO3 Vγ4Vδ1, DP10.7 Vγ4Vδ1 and G7 Vγ9Vδ1 TCR–CD3 complexes exhibit diverse docking topologies towards their ligands^17,20,21^ (Supplementary Fig. 6a). Earlier studies have proposed that γδ TCRs resemble immunoglobins more than αβ TCRs due to their structural similarity to antibody V domains and long CDR3 lengths^2,30,44^. Our study offers insights into this concept (Supplementary Fig. 6b). In contrast to the αβ TCR–CD3 complex, in which its ECD and CPs show structural rigidity^45^, the γδ TCR ECD and CPs resemble the dynamic Fabs and hinge linkers of membrane-bound immunoglobulins, respectively. This considerable conformational flexibility may enable the γδ TCR–CD3 complex to recognize ligands that differ substantially in size and molecular structure, resembling the function of antibodies^2^.

Recent advances revealed that some BTNs or BTN-like proteins regulate specific subsets of γδ TCRs through their HV4 and CDR2 regions in their Vγ chains. These regions, which are entirely germline encoded, probably mediate the non-clonal, innate-like response^7,8,25,46–48^. To date, only human Vγ9 and Vγ4 have been identified for the corresponding BTN or BTN-like ligands^23,25,26,46,48^. Our study demonstrates that dimerization of Vγ5 or Vγ2 domains shields HV4, thereby preventing certain ligands from association (Supplementary Fig. 6c,d). Thus, the potential ligands recognized by Vγ5 or Vγ2 domains are unlikely to interact through the germline-encoded HV4 region. Instead, such binding might occur through the somatic mutation region, such as CDR3. This suggests that dimeric γδ TCRs may exhibit distinct modalities of ligand recognition compared with the monomeric counterparts.

γδ T cells exhibit higher basal activation and stronger signalling compared with those of αβ T cells^49^. This phenomenon coincides with the conformation of CD3ζ in γδ TCR–CD3, which is more similar to that of constitutively active αβ TCR–CD3. Here we demonstrate that the binding of cholesterol to γδ TCR–CD3 exerts an inhibitory role in cell signalling, possibly by modulating the conformation of CD3ζ (Fig. 3). Thus, the conformation of CD3ζ in the γδ TCR–CD3 complex may correlate with strength of TCR signalling. Moreover, mechanical forces have a crucial role in TCR activation^50^. After ligand engagement, the CPs of Cγ1 and Cγ2 may transmit distinct mechanical properties, thereby affecting T cell activation. Consistent with this scenario, the length of CPs has a regulatory role in T cell activation (Fig. 2). However, the exact mechanisms remain to be elucidated and our structures provide a promising starting point for further investigations.

## Methods

### Construct design

The cDNAs encoding the TCR γ chain, δ chain and the four CD3 subunits were codon-optimized and synthesized by Tsingke Biotechnology, and subsequently subcloned into the optimized pCAG vector individually. A synthetic signal peptide (MDMRVPAQLLGLLLLWLSGARC)^53^ was fused at the N terminus of the γ and δ chains, followed by a Flag tag (MDYKDDDDK). Moreover, a twin-Strep II tag was inserted at the C terminus of CD3ζ to facilitate purification (Extended Data Fig. 1a). The coding sequence for the human CD1d soluble region was also codon-optimized and fused with the synthetic signal peptide at its N terminus and 8×His and Avi tags (GLNDIFEAQKIEWHE) at its C terminus. The cDNAs encoding soluble CD1d and human beta-2-microglobulin (β_2_m) were individually subcloned into the expression-optimized pCAG vector. The cDNAs encoding BTN2A1 and BTN3A1 using the same signal peptide at the N terminus were fused with a Flag tag and 8×His tag at its C terminus, respectively. Detailed information regarding these constructs is provided in the Supplementary Information.

### Protein purification of the γδ TCR–CD3 complexes

As previously described^54,55^, 2.4 mg of plasmids (0.4 mg for each plasmid) were pre-incubated with 5 mg of 40 kDa polyethylenimines (PEIs) (Yeasen) in 45 ml of fresh medium (Sino Biological) for 30 min, and then transfected into 1 l of ExpiHEK293F cells (Thermo Fisher Scientific, A14527CN). The transfected cells were cultured at 37 °C under 5% CO_2_ in a Multitron Proshaker (Infors, 120 rpm) for 3 days before collection. The collected cells were homogenized on ice using 25 mM HEPES pH 7.4, 150 mM NaCl containing 1 mM PMSF, 2 μg ml^−1^ pepstatin, 2 μg ml^−1^ aprotinin and 2 μg ml^−1^ leupeptin. The cell membrane was solubilized using 1% (w/v) GDN (Anatrace) at 4 °C for 2 h. Insoluble material and cell debris were removed by centrifugation. The resulting supernatant was applied to anti-Flag M2 affinity resin (Millipore-Sigma), washed with buffer (25 mM HEPES pH 7.4, 150 mM NaCl and 0.02% (w/v) GDN) and eluted using the wash buffer supplemented with 400 μg ml^−1^ of Flag peptide. The eluent from the anti-Flag resin was loaded onto strep-Tactin XT resin (IBA), washed with wash buffer and eluted with 25 mM HEPES pH 7.4, 150 mM NaCl, 0.02% (w/v) GDN and 50 mM d-biotin. The resulting γδ TCR–CD3 complex was concentrated to 1 ml and further fractionated using SEC (Superose 6 increase 10/300, GE Healthcare) in wash buffer. The Vγ9Vδ2 or Vγ5Vδ1 or other TCR–CD3 complex with different Vγ and Vδ domains was purified using similar purification procedures. The SEC fractions were analysed using mouse monoclonal antibodies against Flag tag (CW0287M, CWBIO) and Strep tag (ab76949, Abcam) at a dilution of 1:2,000. The secondary goat anti-mouse antibody (CW0102) was purchased from CWBIO and was used at a dilution of 1: 5,000.

To facilitate the structural determination of the Vγ9Vδ2 TCR–CD3 complex, we made a modification to the detergent used during purification. Specifically, we replaced the previously used detergent, GDN, with LMNG supplemented with CHS. This modification aimed to reduce micelle signals during data processing^56^. The ExpiHEK293F cells expressing the Vγ9Vδ2 TCR–CD3 complex were processed for extraction using 1% (w/v) LMNG with 0.2% (w/v) CHS, and the wash, elution and SEC buffers were changed to that containing 0.005% (w/v) LMNG with 0.001% (w/v) CHS. These changes in the detergent composition improved the quality of the cryo-EM data. This detergent composition was used only for the structural determination of the Vγ9Vδ2 TCR–CD3 complex.

### Preparation of soluble CD1d–α-GalCer complex

To prepare the CD1d–β_2_m complex, CD1d and β_2_m plasmids (each plasmid is 1 mg) were premixed with 5 mg of PEI in 45 ml of fresh medium for 30 min. The mixture was then added to 1 l of cell culture, and the transfected Expi293F cells were cultured at 37 °C under 5% CO_2_ for 5 days before collection. The cells were cleared by centrifugation at 4,000*g* for 10 min. The medium was then concentrated to approximately 100 ml and loaded onto Ni-NTA resin (Qiagen). The resin was extensively washed using buffer (25 mM HEPES, pH 7.4, 150 mM NaCl) with an additional 30 mM imidazole. The CD1d–β_2_m complex was eluted using the same buffer supplemented with 300 mM imidazole and concentrated using a 10 kDa cut-off Centricon (Millipore), and was then further purified by SEC on a column of Superdex 200 increase 10/300 GL. To obtain the lipid-loaded CD1d–α-GalCer complex, α-galactosylceramide (α-GalCer) was incubated with the CD1d–β_2_m complex overnight at a 3:1 molar ratio. Excess lipid was removed by SEC. The resulting protein was frozen in liquid nitrogen and stored at −80 °C for further assays.

### Preparation of soluble BTN2A1

To prepare soluble BTN2A1, a purification procedure similar to soluble CD1d was used. In brief, plasmids encoding soluble BTN2A1 were transfected into Expi293F cells. The medium was concentrated and applied to Ni-NTA resin (Qiagen). The resin was extensively washed, eluted and concentrated. Further purification was achieved by SEC using the Superdex 200 Increase 10/300 GL column. The resulting protein was flash-frozen in liquid nitrogen and stored at −80 °C for subsequent assays.

### Cryo-EM sample preparation and data collection

A 3 μl aliquot of the protein sample was applied to the holey carbon grids (Quantifoil, Au, 300-mesh, R1.2/1.3), which were previously glow discharged with negative charges at 15 mA for 20 s using the Plasma Cleaner PDC-32G-2 (Harrick Plasma). After 3 s of blotting, the grid was quickly plunged into liquid ethane cooled by liquid nitrogen using the Vitrobot Mark IV (Thermo Fisher Scientific) under 100% humidity at 8 °C.

The prepared grids were then transferred to a Krios electron microscope (Thermo Fisher Scientific) operating at 300 kV and equipped with a Gatan K3 Summit detector and GIF Quantum energy filter. Zero-loss video stacks were automatically collected using EPU (Thermo Fisher Scientific) with a slit width of 20 eV on the energy filter and a preset defocus range from −1.0 µm to −2.0 µm in aberration-free image shift mode with a nominal magnification of ×81,000 (ref. ^57^). The image shift was used, and coma was compensated during data collection. The maximum image shift was not more than 10 μm. Each video stack containing 32 frames was exposed to a total electron dose of 50 e^−^ Å^−2^ over 2.56 s.

### Cryo-EM data processing

The flowcharts for data processing of the Vγ9Vδ2 and Vγ5Vδ1 TMα/AAA/EH TCR–CD3 complexes are presented in Supplementary Figs. 2–4, respectively. For the dataset of Vγ9Vδ2 TCR–CD3 complex, the video stacks were motion-corrected using MotionCor2 (ref. ^58^), and dose-weighted micrographs were processed for patch contrast transfer function (CTF) estimation in cryoSPARC (v.4)^59^. Micrographs exhibiting an estimated CTF resolution of less than 4 Å and ice thickness greater than 1.1 were discarded manually. A small subset of randomly selected micrographs was processed for blob picking, particle extraction and multiple rounds of 2D classification, resulting in 2D class averages with high-resolution features. Using these 2D class averages as references, template picking was performed on the whole dataset. Particles extracted from the dataset were binned by a factor of 4 and processed for multiple rounds of 2D classification to remove junk particles. Selected particles were recentred and re-extracted with a 2× binning factor. After 1 round of 2D classification, the particles were then processed for 3D classification through ab Initio reconstruction. The selected particles were recentred and re-extracted without binning and processed for iterative 3D classification against one ‘good’ and three ‘junk’ references (including an empty micelle) until more than 97% of the remaining particles were classified into the good class. Subsequently, selected particles were processed for three parallel ab initio reconstructions (*k* = 3). Particles of good classes were combined with duplicate particles removed, from which a good initial model was generated. This portion of particles was processed for non-uniform (NU) refinement and further local refinement with a tight protein mask, which led to a reconstruction at 3.7 Å global resolution. To retrieve good particles that were classified into bad classes, several rounds of seed-facilitated 3D classification^60^ were performed using the 3.7 Å reconstruction particles as the good seed to retrieve the potential good particles within the original dataset. Through pooling the good particles and further refinement, a final reconstruction at 3.5 Å was obtained. To eliminate noise on the edge and further improve the map quality, the corresponding particle stacks were transferred to RELION using the csparc2star.py script in the UCSF pyem^61^ software suite. A portion of particles from the resultant two classes exhibiting high-resolution features were recovered after 3D classification (skip alignment, *k* = 5) with a soft protein mask in RELION (v.3.1)^62^. Subsequently, this portion of particles was reimported into cryoSPARC (v.4)^59^ and processed for NU refinement. Subsequent global CTF refinement and local refinement with a soft protein mask led to a final reconstruction at a global resolution of 3.4 Å.

For the dataset of Vγ5Vδ1 EH TCR–CD3 complex, the video stacks were motion-corrected using MotionCor2 (ref. ^58^), and dose-weighted micrographs were processed for patch CTF estimation in cryoSPARC (v.4)^59^. Micrographs exhibiting an estimated CTF resolution of less than 4 Å and ice thickness of greater than 1.1 were discarded manually. The reconstruction of Vγ9Vδ2 was low passed to 8 Å to create templates for particle picking. The extracted particles were binned by a factor of 4 and processed for multiple rounds of 2D classification to remove non-protein junk. Well-sorted particles were recentred and re-extracted and binned by a factor of 2. After 2D classification and 3D classification using ab initio reconstruction, the cleaned particles were recentred and re-extracted without binning. After several rounds of 3D classification, a good initial model was obtained and the corresponding particles were processed for NU refinement, leading to a reconstruction at 4.2 Å. Using this portion of particles, the dataset was enlarged by one round of seed-facilitated 3D classification as previously described. After 3D classification, NU refinement and local refinement, a final reconstruction at a global resolution of 3.95 Å was obtained.

For the dataset of the TMα mutant, the video stacks were motion-corrected using MotionCor2 (ref. ^58^), and dose-weighted micrographs were processed for patch CTF estimation in cryoSPARC (v.4)^59^. Micrographs exhibiting an estimated CTF resolution of less than 4 Å and ice thickness of greater than 1.1 and astigmatism of greater than 500 were discarded manually. After template picking, the particles were extracted and binned by a factor of 4 and processed for multiple rounds of 2D classification. Well-sorted particles were recentred and re-extracted and binned by a factor of 2, after which several rounds of 2D classification were performed. Unbinned particles were processed for several rounds of 3D classification and ab initio reconstruction, leading to a good initial model exhibiting a complete TM helix. Subsequent NU refinement at this portion of particles obtained a reconstruction at 3.9 Å resolution. To improve the map quality, the particles were further sorted by hetero refinement at cryoSPARC and then transferred to Relion for further 3D classification (skip alignment, *k* = 4). Particles corresponding to good class were reimported into CryoSPARC. Subsequent NU refinement and local refinement led to a final reconstruction at 3.88 Å global resolution. A similar data processing workflow was applied to the AAA mutant dataset, ultimately resulting in a final reconstruction with a global resolution of 3.81 Å.

To process the dataset of the Vγ5Vδ1 TCR–CD3 complex, the video stacks were motion-corrected with MotionCor2 (ref. ^58^), and dose-weighted micrographs were processed for patch CTF estimation in cryoSPARC (v.4)^59^. Micrographs exhibiting an estimated CTF resolution of less than 4 Å and ice thickness greater than 1.1 were discarded manually. A small subset of micrographs was processed for blob picking, particle extraction and several rounds of 2D classification, resulting in a portion of particles with significant dimer features. This portion of particles was used to train a deep-learning model for identification of particles from all micrographs by Topaz^63^. All of these particles were then extracted by RELION (v.3.1)^62^ with a 4× binning factor. All of the particles were transferred to cryoSPARC (v.4)^59^, and junk was further removed by several rounds of 2D classification. Cleaned-up particles were recentred and re-extracted with a 2× binning factor. This portion of particles was further sorted by several rounds of 2D classification. Well-sorted particles were recentred and re-extracted without binning. Subsequently, the particles were processed for iterative 3D classification against one ‘good’ and three ‘junk’ references until more than 99% of the remaining particles were classified into the good class. Consequently, through a regular 2D and 3D classification procedure, we obtained overall and MPD–TMD cryo-EM reconstructions at nominal resolutions of 9.5 and 5.3 Å, respectively. To solve the structure of the two MPD–TMD regions separately, the particles were processed for further 3D classification and iterative 3D refinement with the monomer map as a reference. Simply, the mask used in the first run of refinement contained both MPD–TMD regions and micelle; then, the signal of the other MPD–TMD region was partially erased in the mask and processed for the next run of refinement; the final mask contained only the protein signal of the MPD–TMD region. After the last run of refinement, a final reconstruction at 4 Å resolution was obtained. To further improve the map quality, the particle stacks were transferred to RELION using the csparc2star.py script in the UCSF pyem^61^ software suite. Particles corresponding to the best class were recovered after 3D classification (skip alignment, *k* = 5) with the 4 Å map as reference and a soft protein mask in RELION (v.3.1)^62^. This portion of particles was reimported into cryoSPARC (v.4)^59^ and processed for local refinement, which led to a reconstruction at 3.9 Å resolution with map quality improved significantly. The data processing procedures of the other MPD–TMD region were similar. For the variable region, the signal of two MPD–TMD regions and micelle was subtracted from relevant particles in cryoSPARC (v.4)^59^. The subtracted particles were recentred and processed for ab initio reconstruction (*k* = 5). Particles corresponding to the class that showed significantly variable region density were pooled and processed for NU refinement followed by global CTF refinement. A final reconstruction at 3.0 Å global resolution was obtained after local refinement with a soft protein mask.

All final maps were post-processed using DeepEMhancer^64^ to enhance the protein signal, yielding high-quality noise-reduction and signal-enhancement maps. The resolution of the reconstruction was determined on the basis of the gold standard Fourier shell correlation (FSC) 0.143 criterion in cryoSPARC (v.4)^59^, Relion (v.3.1)^62^ or using Phenix.mtriage^65^. 3D FSC analysis for the final maps was performed on the Remote 3DFSC Processing Server^66^ (https://3dfsc.salk.edu/). All of the map figures were generated in ChimeraX^51^ or Chimera^67^.

### Model building and refinement

DeepEMhancer maps and *B*-factor-sharpened maps were used to aid model building and the DeepEMhancer-sharpened maps were used for figure preparation. The initial model of the Vγ9Vδ2 TCR–CD3 complex was built using MDFF^68^ with the reported TCR–CD3 structure (PDB: 7FJD)^40^ as an initial template. The ECD models of CD3ε′δ and CD3εγ from the reported TCR–CD3 structure were docked in the cryo-EM map of the Vγ9Vδ2 TCR–CD3 complex. The model of the Vγ5Vδ1 TCR ECD was built on the basis of the reported structure of 9C2 γδ TCR (PDB: 4LFH)^6^. The molecular model of TMα/AAA/EH TCR–CD3 complexes were built using the model of Vγ9Vδ2 TCR–CD3 complex as an initial template. These models were manually checked and were then refined using PHENIX^65^ and COOT^69^, and all structural refinements were performed in PHENIX in real space, with secondary structure and geometry restraints^65^. Overfitting of the models was monitored by refining the model against one of the two independent half maps from the gold-standard refinement approach and testing the refined model against the other map^70^. The structures were validated through examination of the Molprobity^71^ scores and statistics of the Ramachandran plots (Extended Data Tables 1–3).

The composite map of the Vγ5Vδ1 TCR–CD3 complex was generated by docking the cryo-EM maps of the ECD, MPD_I_–TMD_I_ and MPD_II_–TMD_II_ into the overall map through the transitional MPD–TMD map (Extended Data Fig. 3e–i). The models of the Vγ5Vδ1 TCR complex for the MPD_I_–TMD_I_ and MPD_II_–TMD_II_ regions were obtained by docking the molecular models of the Vγ9Vδ2 TCR–CD3 complex into the respective cryo-EM maps (Extended Data Fig. 3d). The overall model of the Vγ5Vδ1 TCR complex was constructed by docking the models of the Vγ9Vδ2 TCR–CD3 complex and the Vγ5Vδ1 TCR–CD3 ECD into the composite overall map.

### Quantification and identification of cholesterol 3-sulfate and cholesterol by LC–MS/MS

A total of 200 μl protein solution was reduced with 5 mM dithiothreitol at 37 °C for 30 min and alkylated with 20 mM idoacetamide at 25 °C for 30 min. The protein samples were then digested with trypsin (Promega) at an enzyme-to-protein ratio of 1:50 (w/w) at 37 °C overnight. The digested solution was extracted with methyl *tert*-butyl ether by vortex and sonication. The supernatants were collected and lyophilized under vacuum. Extracted lipids were dissolved in 200 μl of methanol for LC–MS/MS analysis.

For detection of cholesterol 3-sulfate, a triple-quadrupole mass spectrometer (SCIEX 6500+) equipped with an electrospray ionization source was used in the negative mode. Compounds were measured by multiple reaction monitoring with optimized cone voltage and collision energy. Cholesterol 3-sulfate signals were monitored by ion transitions at *m*/*z* 465.4 > 96.9 and 465.4 > 79.9. The samples were separated on the Acquity UPLC BEH C18 column (50 mm×2.1 mm, 1.7 μm) at 40 °C. The mobile phase consisted of 20 mM ammonium acetate in water (A) and methanol (B) at a flow rate of 0.4 ml min^−1^. The isocratic elution with mobile phase A and mobile phase B at ratios of 1:9. The content of cholesterol 3-sulfate was calculated by using peak area of standard. MS/MS spectra were collected using the quadrupole time of flight mass spectrometer (Agilent 6545) connected to an ultra-performance liquid chromatography system (Agilent 1290).

For detection of cholesterol, a triple-quadrupole mass spectrometer (SCIEX 6500+) equipped with an atmospheric pressure chemical ionization source (APCI) was used in the positive mode. Compounds were measured by multiple reaction monitoring with optimized cone voltage and collision energy. Cholesterol signals were monitored by ion transitions at *m*/*z* 369.3([M+H-H_2_O]^+^) > 147.3 and 369.3 > 161.2. The samples were separated on the Acquity UPLC BEH C18 column (50 mm × 2.1 mm, 1.7 μm) at 40 °C. The mobile phase consisted of 0.1% formic acid in water (A) and 0.1% formic acid in methanol (B) at a flow rate of 0.6 ml min^−1^. The gradient was as follows: mobile phase B 90% to 100% in 6 min, held at 100% for 2 min, then 100% to 90% in 0.1 min, held at 100 for 1.9 min. The content of cholesterol was calculated by using the peak area of standard. MS/MS spectra were collected using the quadrupole time of flight mass spectrometer (Agilent 6545) connected to an ultra-performance liquid chromatography system (Agilent 1290).

### Generation of stable cell lines

TCRγ5 chain with or without EH mutation and TCRδ1 chain were separated by a P2A ribosome-skipping site (ATNFSLLKQAGDVEENPGP) and cloned into the lentiCRISPR v2 vector^72^ with a spleen focus-forming virus (SFFV) promoter^73^ and a mGreenLantern^74^ at the C terminus. TCRγ9 chain and TCRδ2 chain were cloned using the same strategy. The lentiviral transfer plasmids encoding WT Vγ5Vδ1, WT Vγ9Vδ2 and EH mutant Vγ5Vδ1 TCRs, along with the packaging vectors psPAX2 and pMD2.G, were co-transfected into Lenti-X 293T (Clontech, 632180) cells. The cells were grown in DMEM (Gibco) supplemented with 10% (v/v) FBS (Gibco), penicillin G (100 U ml^−1^) and streptomycin (100 μg ml^−1^; Sino Biological) at 37 °C in a water-saturated atmosphere containing 5% CO_2_. Then, 3 days after transfection, the supernatants were collected and cell debris was cleared by centrifugation at 1,000*g* and 0.45 μm filtered (Beyotime). Then, 80 μg ml^−1^ protamine sulfate (Macklin) and 80 μg ml^−1^ chondroitin sulfate C (Macklin) were added to concentrate the lentivirus, as previously reported^75^. Next, 1 × 10^6^ Jurkat-76 cells^76,77^ in 1 ml RPMI 1640 medium with an additional 5 μg ml^−1^ polybrene (Sigma-Aldrich) were transduced with 1 ml lentiviruses. Then, 24 h later, 1 × 10^6^ of these cells were retransduced with the 1 ml lentiviruses. The cells with similar green fluorescence intensities were sorted using flow cytometry to ensure similar WT Vγ5Vδ1 and EH mutant Vγ5Vδ1 TCR expression levels. Furthermore, to identify the surface expression level of WT and EH- and R120Q-mutant Vγ5Vδ1 TCRs, the anti-Flag antibodies (ABclonal, AE116) were used at a dilution of 1: 500 for 30 min on ice.

For APCs, full length *CD1D* was cloned into lentiCRISPR v2 vector with a SFFV promoter and a mCherry at the C terminus. *BTN2A1* and *BTN3A1*, separated by two P2A, were cloned into the same vector. Moreover, ZIM3–dCas9 (ref. ^52^) was used as a negative control and was also cloned into the vector. Lentiviruses encoding CD1d, BTN2A1-BTN3A1 and ZIM3–dCas9 (ref. ^52^) were produced using the same procedure mentioned above. The mCherry^+^ K562 cells (ATCC, CCL243) were selected using flow cytometry.

### T cell activation assay

To detect the upregulation of CD69, approximately 1 × 10^6^ Jurkat cells expressing the TCRs of interest were seeded per well of a six-well plate. The Jurkat cells were then co-cultured with K562 cells expressing CD1d, BTN2A1–BTN3A1 or ZIM3–dCas9 (ref. ^52^) at a 1:1 ratio for 24 or 48 h. Hydroxymethylbut-2-enyl pyrophosphate (Sigma-Aldrich) was added at a final concentration of 1 μM when Vγ9Vδ2 TCR^+^ Jurkat cells were cultured with K562 cells. As positive controls, WT or mutant Vγ5Vδ1 TCR^+^ Jurkat cells were treated with anti-CD3 (Invitrogen, 16-0037-81) and anti-CD28 antibodies (Invitrogen, 16-0289-81) at a dilution of 1:100 for 24 h. The cells were collected and washed twice in fluorescence-activated cell sorting (FACS) buffer consisting of PBS supplemented with 2% (v/v) FBS. The cells were then incubated with anti-human CD69-APC (Sino Biological, 11150-MM06-A) and CD3-PE-Cyanine7 dye antibodies (BD Pharmingen, 552127) at a dilution of 1:500 for 30 min on ice. Next, the cells were washed again in FACS buffer. Before the FACS analysis, 4′,6-diamidino-2-phenylindole (Solarbio) was added to the samples at a final concentration of 0.1 µg ml^−1^. The percentage of CD69 expression of each group was analysed using the Cyto-FLEX LX-5L2 (Beckman) system. Resulting data were analysed using FlowJo (v.10.8.1; Tree Star, BD Biosciences). A representative gating strategy is given in Supplementary Fig. 1b.

### Tetramerization of CD1d–α-GalCer complex and BTN2A1

The CD1d–α-GalCer or BTN2A1 tetramer was generated according to a previously reported method^78^. In brief, the cDNAs of the soluble CD1d or BTN2A1 protein with an Avi tag and the biotin ligase BirA^79^ were individually cloned into an optimized pCAG vector and co-expressed in Expi293F cells. After 24 h of transduction, the medium was supplemented with 100 μM biotin (Fluka) and incubated for 5 days at 37 °C with 5% CO_2_. The in vivo biotinylated CD1d–β_2_m complexes or BTN2A1 were then purified as previously described. To load the lipid, α-GalCer was incubated with the biotinylated CD1d–β_2_m complex overnight and the excess lipid was removed by SEC. The CD1d–α-GalCer tetramers were generated by incubating the biotinylated CD1d–α-GalCer complex with Streptavidin-APC (Solarbio) at 4 °C for 6 h. The CD1d–α-GalCer or BTN2A1 tetramers can be stored at 4 °C for up to 1 month.

### Flow cytometry staining and analysis

Jurkat cells expressing WT Vγ5Vδ1, EH mutant Vγ5Vδ1 or WT Vγ9Vδ2 TCRs were washed three times with FACS buffer before surface staining. For tetramer staining, 2 × 10^6^ cells were suspended in 200 μl of FACS buffer, incubated with CD1d–α-GalCer or BTN2A1 tetramers at a final concentration of 10 μg ml^−1^ at 37 °C for 30 min. FACS data acquisition was performed using the Cyto-FLEX LX-5L2 (Beckman) and the data were analysed using FlowJo software (Tree Star).

### Preparation of ALOD4 and ALOD4 non-binding mutant

ALOD4 and the ALOD4 non-binding mutant were expressed in *E. coli* BL21 (DE3) cells. Recombinant protein expression was induced by the addition of 1 mM IPTG at 18 °C. After overnight induction, the cells were collected by centrifugation and resuspended in buffer containing 25 mM Tris-HCl (pH 8.0) and 150 mM NaCl. The buffer was supplemented with 1 mM of the protease inhibitor PMSF to ensure protein stability. Cell lysis was achieved by sonication, and the resulting lysate was cleared of cellular debris by centrifugation at 30,000*g* for 40 min at 4 °C. The lysate was initially purified using amylose beads from GE Healthcare. The beads were washed to remove any unbound proteins, and the bound proteins were processed for cleavage by drICE^80^ at 4 °C for 2 h. This cleavage step was used to remove the maltose-binding protein tag. The cleaved ALOD4 and ALOD4 non-binding mutant proteins were eluted from the amylose resin and subsequently fractionated using SEC with the Superdex 200 Increase 10/300 column from GE Healthcare. The fractionation was performed in PBS buffer.

### ALOD4 assay

A total of 3 × 10^5^ Vγ5Vδ1 or Vγ9Vδ2 TCR-expressing cells was collected and suspended in 500 μl of FACS buffer. To sequester cell plasma membrane cholesterol levels, a treatment was carried out at 37 °C using 0.5 μM of either ALOD4 or the ALOD4 nonbinding mutant for a duration of 12 h. Subsequently, the percentage of CD69 expression in the Vγ5Vδ1 or Vγ9Vδ2 TCR-expressing cells from each experimental group was analysed using the Cyto-FLEX LX-5L2 flow cytometer (Beckman).

### FLIM–FRET measurement and analysis

Full-length TCRγ or its variants were fused to CFP (moxCerulean3 (ref. ^81^)) and YFP (mGold^82^), serving as the FRET donor and acceptor, respectively. GGGGGS linkers were inserted between the TCRγ or its variants and the fluorescent proteins. TCRδ and TCRγ or its variants were separated by P2A ribosome-skipping sites. The full-length CD3 subunits were tandemly linked according to the following order: CD3γ–T2A–CD3ε–P2A–CD3δ–E2A–CD3ζ. In HEK293T cells, the donor plasmids and the CD3 subunits were transfected as the donor group; both donor and acceptor plasmids at a DNA mass ratio of 10:1 were co-transfected with the CD3 plasmids as the donor + acceptor group. FLIM–FRET requires a minimum of 1,000 photons per pixel during donor acquisitions. With a 1:1 transfection ratio, the intensity of the donor fluorescence was too weak to meet this requirement. However, by increasing the donor-to-acceptor ratio to 10:1, the intensity of CFP fluorescence was strong enough to obtain an adequate number of photons for accurate donor lifetime measurements.

Fluorescence lifetime measurements of the donor fluorophore, both in the absence and presence of the acceptor fluorophore, were conducted using the Leica STELLARIS8 microscope equipped with a confocal scan head featuring field-programmable gate array electronics, pulsed laser excitation at 448 nm and fast spectral single-photon counting detectors (HyD family). Lifetime images were acquired at a rate of 400 Hz using XYZ acquisition mode, with a high spatial resolution of 512 px × 512 px. To ensure reliable lifetime data, photons from the entire image were recorded, with a minimum of 1,000 photons per pixel captured for both donor and acceptor acquisitions. The average photon arrival per pixel was then used to generate FLIM images. Cells expressing moxCerulean3 and mGold were selected, and the average lifetime within the cell membrane was determined for the cell of interest. The time-correlated single-photon counting decay curves were fitted using the multi-exponential donor fit model, enabling the calculation of the average lifetime based on multiple cells. The amplitude of each lifetime component was used to indicate its proportion, and the intensity-weighted mean *τ* was calculated as the average fluorescence lifetime. The FRET efficiency (*E*) was determined using the following equation:

$$E=1-\frac{{\tau }_{{\rm{AvAmp}}}}{{\tau }_{{\rm{D}}}}$$

where *τ*_D_ represents the lifetime value of the unquenched donor lifetime, and *τ*_AvAmp_ represents the mean decay time (amplitude-weighted average lifetime).

The FLIM–FRET data were analysed using the LAS X Life Science Microscope Software Platform (Leica).

### Reporting summary

Further information on research design is available in the Nature Portfolio Reporting Summary linked to this article.

## Online content

Any methods, additional references, Nature Portfolio reporting summaries, source data, extended data, supplementary information, acknowledgements, peer review information; details of author contributions and competing interests; and statements of data and code availability are available at 10.1038/s41586-024-07439-4.

## Supplementary information

Supplementary Information Supplementary Methods, Supplementary Figs. 1–9 and Supplementary Table 1.

Reporting Summary

## Source data

Source Data Figs. 2, 3 and 5 and Source Data Extended Data Fig. 9

## Figures and Tables

**Extended Data Table 1:**
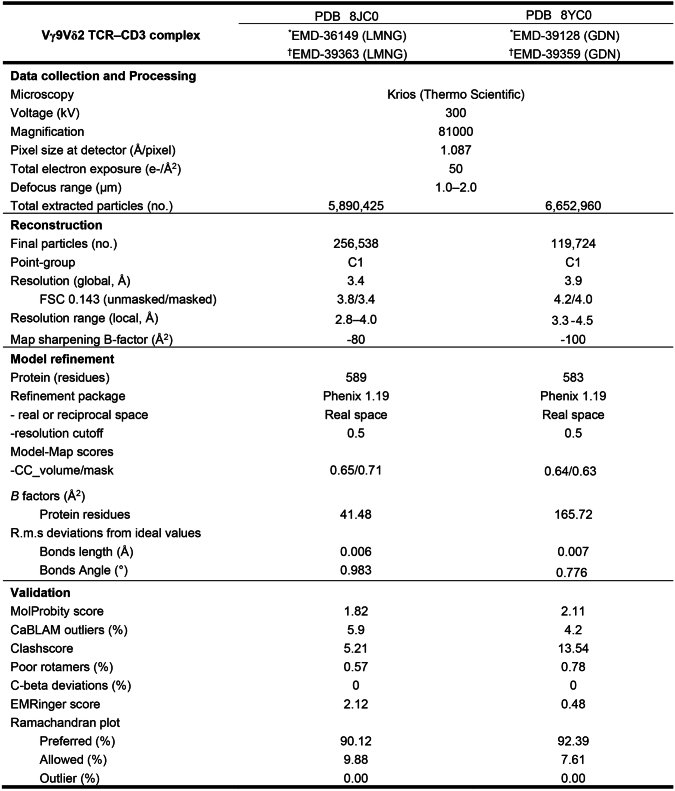
Statistics of cryo-EM analysis of the human Vγ9Vδ2 complex in LMNG and GDN detergent micelles

^*^The cryo-EM maps sharpened by DeepEMhancer. ^†^The cryo-EM maps sharpened by B-factor.

**Extended Data Table 2:**
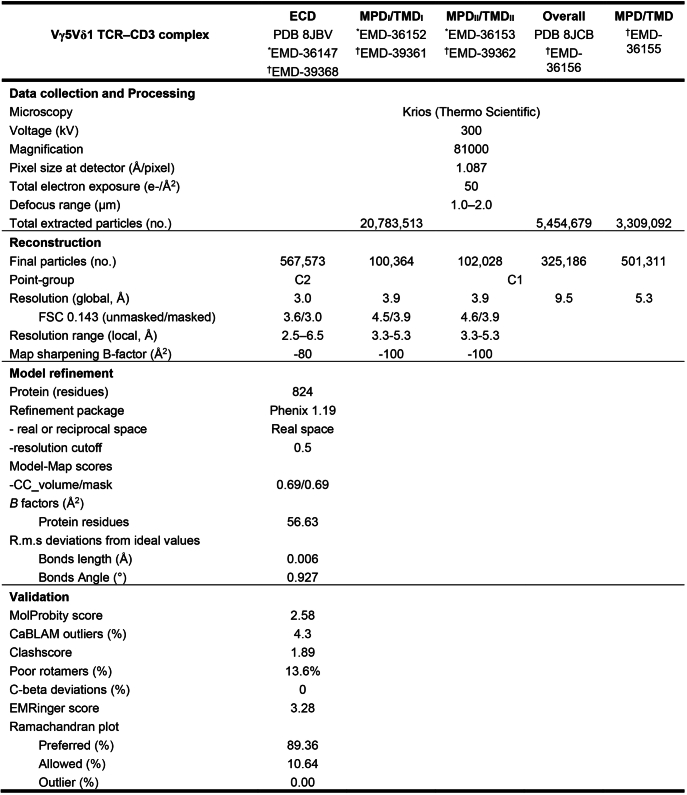
Statistics of cryo-EM analysis of the human Vγ5Vδ1 TCR–CD3 complex

^*^The cryo-EM maps sharpened by DeepEMhancer. ^†^The cryo-EM maps sharpened by B-factor.

**Extended Data Table 3:**
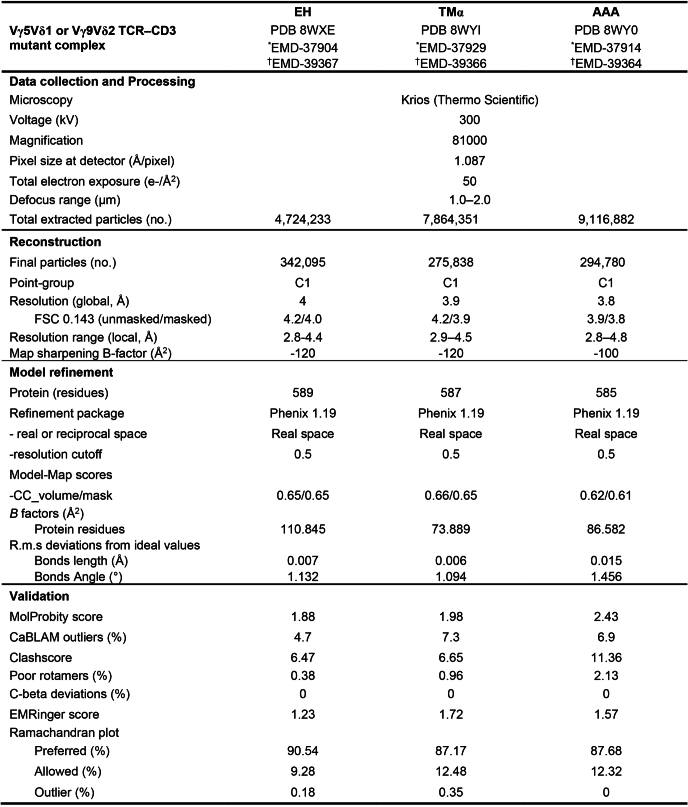
Statistics of cryo-EM analysis of the mutant Vγ5Vδ1 and Vγ9Vδ2 TCR–CD3 complex

^*^The cryo-EM maps sharpened by DeepEMhancer. ^†^The cryo-EM maps sharpened by B-factor.

## Data Availability

The atomic coordinates for Vγ9Vδ2 and Vγ5Vδ1 TCR–CD3 complexes have been deposited at the PDB under accession codes 8JC0 (WT/LMNG), 8YC0 (WT/GDN), 8WYI (TMα), 8WY0 (AAA), 8JCB (overall), 8JBV (ECD) and 8WXE (EH), respectively. For Vγ9Vδ2 TCR–CD3 complexes, the cryo-EM maps sharpened by DeepEMhancer have been deposited at the Electron Microscopy Data Bank (EMDB) under accession codes EMD-36149 (WT/LMNG), EMD-39128 (WT/GDN), EMD-37929 (TMα) and EMD-37914 (AAA). The cryo-EM maps sharpened using *B*-factor have been deposited at the EMDB under accession codes EMD-39363 (WT/LMNG), EMD-39359 (WT/GDN), EMD-39366 (TMα) and EMD-39364 (AAA). For Vγ5Vδ1 TCR–CD3 complexes, the cryo-EM maps sharpened by DeepEMhancer of have been deposited at the EMDB under accession codes EMD-36152 (MPD_I_–TMD_I_), EMD-36153 (MPD_II_–TMD_II_), EMD-36147 (ECD) and EMD-37904 (EH). The cryo-EM maps sharpened using *B*-factor have been deposited at the EMDB under accession codes EMD-36156 (overall), EMD-36155 (MPD–TMD), EMD-39361 (MPD_I_–TMD_I_), EMD-39362 (MPD_II_–TMD_II_), EMD-39368 (ECD) and EMD-39367 (EH). To prepare figures, we used structural information from the PDB (7FJD, 1HXM, 7FJE, 4LHU, 7RYN, 4MNG, 6MWR, 7PHR, 7XQ8, 7WSO, 8DFW, 8IGT, 8DFX, 7RYL and 4F9P). All data are available in the Article and the Supplementary Information. All materials are available from the corresponding authors on reasonable request. Source data are provided with this paper.

## Code availability

No code was used for this study.
