# Supplementary material for: Structures of human γδ T cell receptor–CD3 complex
Source: Nature. 2024 Apr 24;630(8015):222–9. doi: 10.1038/s41586-024-07439-4 (PMC11153141; doi:10.1038/s41586-024-07439-4)
Supplement: Supplementary file 1 — Supplementary Methods, Supplementary Figs. 1–9 and Supplementary Table 1. [file 41586_2024_7439_MOESM1_ESM.pdf]

nature portfolio

https://doi.org/10.1038/s41586-024-07439-4

Supplementary information

Structures of human γδ T cell receptor–CD3 complex

In the format provided by the authors and unedited

Supplementary Information

Structures of human γδ T cell receptor–CD3 complex

Weizhi Xin[1,2,4], Bangdong Huang[1,2,4], Ximin Chi[1,2,3,4], Yuehua Liu[1,2], Mengjiao Xu[1,2], Yuanyuan Zhang[1,2], Xu Li[1,2], Qiang Su[1,2,4]*, Qiang Zhou[1,2]*

[1]Research Center for Industries of the Future, Center for Infectious Disease Research, Zhejiang Key Laboratory of Structural Biology, School of Life Sciences, Westlake University; Institute of Biology, Westlake Institute for Advanced Study, Hangzhou, Zhejiang Province, China

[2]Westlake Laboratory of Life Sciences and Biomedicine, Hangzhou, Zhejiang Province, China

[3]Present address: State Key Laboratory of Cellular Stress Biology, Innovation Center for Cell Signaling Network, School of Life Science, Xiamen University, Xiamen, Fujian Province, China.

[4]These authors contributed equally to this work.

*Corresponding author. Email: zhouqiang@westlake.edu.cn (Q.Z.); suqiang@smart.org.cn (Q.S.)

Table of contents

Supplementary Methods | Amino acid sequences of the constructs used in this study. 2

Supplementary Fig. 1 | Gating strategy used in the CD69 expression analysis and functional validation of Jurkat-76 cells stably expressing γδ TCR of interest. 7

Supplementary Fig. 2 | The flowchart for cryo-EM data processing of the human Vγ9Vδ2 TCR–CD3 complex in LMNG or GDN detergent micelles. 8

Supplementary Fig. 3 | The flowchart for cryo-EM data processing of the human Vγ5Vδ1 TCR–CD3 complex. 9

Supplementary Fig. 4 | The flowchart for cryo-EM data processing of the mutant Vγ9Vδ2 and mutant Vγ5Vδ1 TCR–CD3 complexes. 10

Supplementary Fig. 5 | Vγ5Vδ1 TCR–CD3 complex recognizes CD1d–β2m in a bivalent binding manner. 11

Supplementary Fig. 6 | Different ligand recognition modalities of the monomeric and dimeric γδ TCRs, αβ TCRs and BCRs. 12

Supplementary Fig. 7 | Uncropped images for the Extended Data Figure 1c. 13

Supplementary Fig. 8 | Uncropped images for the Extended Data Figure 1d. 14

Supplementary Fig. 9 | Uncropped images for the Extended Data Figure 8b. 15

Supplementary Table S1 | The interactions within the $V\gamma5_{I}$–$V\gamma5_{II}$ interface. 16

Supplementary Methods

Amino acid sequences of the constructs used in this study

TCRδ1:

MDMRVPAQLLGLLLLWLSGARCMDYKDDDDKGGSETGAQKVTQAQSSVSM
PVRKAVTLNCLYETSWWSYYIFWYKQLPSKEMIFLIRQGSDEQNAKSGRYSV
NFKKAAKSVALTISALQLEDSAKYFCALGDPGGLNTDKLIFGKGTRVTVEPRS
QPHTKPSVFVMKNGTNVACLVKEFYPKDIRINLVSSKKITEFDPAIVISPSGKYN
AVKLGKYEDSNSVTCSVQHDNKTVHSTDFEVKTDSTDHVKPKETENTKQPSK
SCHKPKAIVHTEKVNMMSLTVLGLRMLFAKTVAVNFLLTAKLFFL

TCRγ5:

MDMRVPAQLLGLLLLWLSGARCMDYKDDDDKGGSETGSSNLEGGTKSVTRP
TRSSAEITCDLTVINAFYIHWYLHQEGKAPQRLLYYDVSNSKDVLESGLSPGK
YYTHTPRRWSWILILRNLIENDSGVYYCATWDRGNPKTHYYKKLFGSGTTLV
VTDKQLDADVSPKPTIFLPSIAETKLQKAGTYLCLLEKFFPDVIKIHWQEKKSN
TILGSQEGNTMKTNDTYMKFSWLTVPEKSLDKEHRCIVRHENNKNGVDQEIIF
PPIKTDVITMDPKDNCSKDANDTLLLQLTNTSAYYMYLLLLLKSVVYFAIITCC
LLRRTAFCCNGEKS

TCRδ2:

MDMRVPAQLLGLLLLWLSGARCMDYKDDDDKGGSETGAIELVPEHQTVPVSI
GVPATLRCSMKGEAIGNYYINWYRKTQGNTMTFIYREKDIYGPGFKDNFQGD
IDIAKNLAVLKILAPSERDEGSYYCACDTLGMGGEYTDKLIFGKGTRVTVEPR
SQPHTKPSVFVMKNGTNVACLVKEFYPKDIRINLVSSKKITEFDPAIVISPSGKY
NAVKLGKYEDSNSVTCSVQHDNKTVHSTDFEVKTDSTDHVKPKETENTKQPS
KSCHKPKAIVHTEKVNMMSLTVLGLRMLFAKTVAVNFLLTAKLFFL

TCRγ9:

MDMRVPAQLLGLLLLWLSGARCMDYKDDDDKGGSETGAGHLEQPQISSTKT
LSKTARLECVVSGITISATSVYWYRERPGEVIQFLVSISYDGTVRKESGIPSGKF
EVDRIPETSTSTLTIHNVEKQDIATYYCALWEAQQELGKKIKVFGPGTKLIITDK
QLDADVSPKPTIFLPSIAETKLQKAGTYLCLLEKFFPDVIKIHWQEKKSNTILGS
QEGNTMKTNDTYMKFSWLTVPEKSLDKEHRCIVRHENNKNGVDQEIIFPPIKT
DVITMDPKDNCSKDANDTLLLQLTNTSAYYMYLLLLLKSVVYFAIITCCLLRR
TAFCCNGEKS

CD3ε:

MQSGTHWRVLGLCLLSVGVWGQDGNEEMGGITQTPYKVSISGTTVILTCPQY
PGSEILWQHNDKNIGGDEDDKNIGSDEDHLSLKEFSELEQSGYYVCYPRGSKP
EDANFYLYLRARVCENCMEMDVMSVATIVIVDICITGGLLLLVYYWSKNRKA
KAKPVTRGAGAGGRQRGQNKERPPPVPNPDYEPIRKGQRDLYSGLNQRRI

CD3γ:

MEQGKGLAVLILAIILLQGTLAQSIKGNHLVKVYDYQEDGSVLLTCDAEAKNI
TWFKDGKMIGFLTEDKKKWNLGSNAKDPRGMYQCKGSQNKSKPLQVYYRM
CQNCIELNAATISGFLFAEIVSIFVLAVGVYFIAGQDGVRQSRASDKQTLLPND
QLYQPLKDREDDQYSHLQGNQLRRN

CD3δ:

MEHSTFLSGLVLATLLSQVSPFKIPIEELEDRVFVNCNTSITWVEGTVGTLLSDI
TRLDLGKRILDPRGIYRCNGTDIYKDKESTVQVHYRMCQSCVELDPATVAGII
VTDVIATLLLALGVFCFAGHETGRLSGAADTQALLRNDQVYQPLRDRDDAQY
SHLGGNWARNK

CD3ζ:

MKWKALFTAAILQAQLPITEAQSFGLLDPKLCYLLDGILFIYGVILTALFLRVK
FSRSADAPAYQQGQNQLYNELNLGRREEYDVLDKRRGRDPEMGGKPQRRKN
PQEGLYNELQKDKMAEAYSEIGMKGERRRGKGHDGLYQGLSTATKDTYDAL
HMQALPPRAAAWSHPQFEKGGGSGGGSGGSAWSHPQFEK

Soluble CD1d:

MDMRVPAQLLGLLLLWLSGARCEVPQRLFPLRCLQISSFANSSWTRTDGLAW
LGELQTHSWSNDSDTVRSLKPWSQGTFSDQQWETLQHIFRVYRSSFTRDVKE
FAKMLRLSYPLELQVSAGCEVHPGNASNNFFHVAFQGKDILSFQGTSWEPTQE
APLWVNLAIQVLNQDKWTRETVQWLLNGTCPQFVSGLLESGKSELKKQVKP
KAWLSRGPSPGPGRLLLVCHVSGFYPKPVWVKWMRGEQEQQGTQPGDILPN
ADETWYLRATLDVVAGEAAGLSCRVKHSSLEGQDIVLYWGGSHHHHHHHH

CD1d:

MDMRVPAQLLGLLLLWLSGARCEVPQRLFPLRCLQISSFANSSWTRTDGLAW
LGELQTHSWSNDSDTVRSLKPWSQGTFSDQQWETLQHIFRVYRSSFTRDVKE
FAKMLRLSYPLELQVSAGCEVHPGNASNNFFHVAFQGKDILSFQGTSWEPTQE
APLWVNLAIQVLNQDKWTRETVQWLLNGTCPQFVSGLLESGKSELKKQVKP
KAWLSRGPSPGPGRLLLVCHVSGFYPKPVWVKWMRGEQEQQGTQPGDILPN
ADETWYLRATLDVVAGEAAGLSCRVKHSSLEGQDIVLYWGGSYTSMGLIALA
VLACLLFLLIVGFTSRFKRQTSYQGVL

β_2m:

MDMRVPAQLLGLLLLWLSGARCIQRTPKIQVYSRHPAENGKSNFLNCYVSGF
HPSDIEVDLLKNGERIEKVEHSDLSFSKDWSFYLLYYTEFTPTEKDEYACRVN
HVTLSQPKIVKWDRDM

BTN2A1:

MDMRVPAQLLGLLLLWLSGARCQFIVVGPTDPILATVGENTTLRCHLSPEKNA
EDMEVRWFRSQFSPAVFVYKGGRERTEEQMEEYRGRTTFVSKDISRGSVALVI
HNITAQENGTYRCYFQEGRSYDEAILHLVVAGLGSKPLISMRGHEDGGIRLECI
SRGWYPKPLTVWRDPYGGVAPALKEVSMPDADGLFMVTTAVIIRDKSVRNMS
CSINNTLLGQKKESVIFIPESFMPSVSPCAVALPIIVVILMIPIAVCIYWINKLQKE
KKILSGEKEFERETREIALKELEKERVQKEEELQVKEKLQEELRWRRTFLHAV
DVVLDPDTAHPDLFLSEDRRSVRRCPFRHLGESVPDNPERFDSQPCVLGRESF
ASGKHYWEVEVENVIEWTVGVCRDSVERKGEVLLIPQNGFWTLEMHKGQY
RAVSSPDRILPLKESLCRVGVFLDYEAGDVSFYNMRDRSHIYTCPRSAFSVPVR
PFFRLGCEDSPIFICPALTGANGVTVPEEGLTLHRVGTHQSLGSSGMDYKDDD
DK

BTN3A1 :

MDMRVPAQLLGLLLLWLSGARCQFSVLGPSGPILAMVGEDADLPCHLFPTMS
AETMELKWVSSSLRQVVNVYADGKEVEDRQSAPYRGRTSILRDGITAGKAAL
RIHNVTASDSGKYLCYFQDGDFYEKALVELKVAALGSDLHVDVKGYKDGGI
HLECRSTGWYPQPQIQWSNNKGENIPTVEAPVVADGVGLYAVAASVIMRGSS
GEGVSCTIRSSLLGLEKTASISIADPFFRSAQRWIAALAGTLPVLLLLLGGAGYF
LWQQQEEKKTQFRKKKREQELREMAWSTMKQEQSTRVKLLEELRWRSIQYA
SRGERHSAYNEWKKALFKPADVILDPKTANPILLVSEDQRSVQRAKEPQDLPD
NPERFNWHYCVLGCESFISGRHYWEVEVGDRKEWHIGVCSKNVQRKGWVK
MTPENGFWTMGLTDGNKYRTLTEPRTNLKLPKPPKKVGVFLDYETGDISFYN
AVDGSHIHTFLDVSFSEALYPVFRILTLEPTALTICPAGSSGHHHHHHHH

ECD BTN2A1:

MDMRVPAQLLGLLLLWLSGARCQFIVVGPTDPILATVGENTTLRCHLSPEKNA
EDMEVRWFRSQFSPAVFVYKGGRERTEEQMEEYRGRTTFVSKDISRGSVALVI
HNITAQENGTYRCYFQEGRSYDEAILHLVVAGLGSKPLISMRGHEDGGIRLECI

SRGWYPKPLTVWRDPYGGVAPALKEVSMPDADGLFMVTTAVIIRDKSVRNMS
CSINNTLLGQKKESVIFIPESFMPSVSGTGSGSGGLNDIFEAQKIEWHEHHHHH
H

TCRδ2 (Cα/CPα):

MDMRVPAQLLGLLLLWLSGARCMDYKDDDDKGGSETGAIELVPEHQTVPVSI
GVPATLRCSMKGEAIGNYYINWYRKTQGNTMTFIYREKDIYGPGFKDNFQGD
IDIAKNLAVLKILAPSERDEGSYYCACDTLGMGGEYTDKLIFGKGTRVTVEPRI
QNPDPAVYQLRDSKSSDKSVCLFTDFDSQTNVSQSKDSDVYITDKTVLDMRS
MDFKSNSAVAWSNKSDFACANAFNNSIIPEDTFFPSPESSCDVKLVEKSFETDT
NLNMMSLTVLGLRMLFAKTVAVNFLLTAKLFFL

TCRγ9 (Cβ2/CPβ2):

MDMRVPAQLLGLLLLWLSGARCMDYKDDDDKGGSETGAGHLEQPQISSTKT
LSKTARLECVVSGITISATSVYWYRERPGEVIQFLVSISYDGTVRKESGIPSGKF
EVDRIPETSTSTLTIHNVEKQDIATYYCALWEAQQELGKKIKVFGPGTKLIITDL
KNVFPPKVAVFEPSEAEISHTQKATLVCLATGFYPDHVELSWWVNGKEVHSG
VSTDPQPLKEQPALNDSRYCLSSRLRVSATFWQNPRNHFRCQVQFYGLSENDE
WTQDRAKPVTQIVSAEAWGRADCGFTSESYQQLTNTSAYYMYLLLLLKSVV
YFAIITCCLLRRTAFCCNGEKS

TCRγ9 (Cγ2):

MDMRVPAQLLGLLLLWLSGARCMDYKDDDDKGGSETDAGHLEQPQISSTKT
LSKTARLECVVSGITISATSVYWYRERPGEVIQFLVSISYDGTVRKESGIPSGKF
EVDRIPETSTSTLTIHNVEKQDIATYYCALWEAQQELGKKIKVFGPGTKLIITDK
QLDADVSPKPTIFLPSIAETKLQKAGTYLCLLEKFFPDVIKIHWQEKKSNTILGS
QEGNTMKTNDTYMKFSWLTVPEKSLDKEHRCIVRHENNKNGVDQEIIFPPIKT
DVTTVDPKYNYSKDANDVITMDPKDNCSKDANDTLLLQLTNTSAYYMYLLL
LLKSVVYFAIITCCLLRRTAFCCNGEKS

TCRγ5 (Cγ2):

MDMRVPAQLLGLLLLWLSGARCMDYKDDDDKGGSETGSSNLEGGTKSVTRP
TRSSAEITCDLTVINAFYIHWYLHQEGKAPQRLLYYDVSNSKDVLESGLSPGK
YYTHTPRRWSWILILRNLIENDSGVYYCATWDRGNPKTHYYKKLFGSGTTLV
VTDKQLDADVSPKPTIFLPSIAETKLQKAGTYLCLLEKFFPDVIKIHWQEKKSN
TILGSQEGNTMKTNDTYMKFSWLTVPEKSLDKEHRCIVRHENNKNGVDQEIIF
PPIKTDVTTVDPKYNYSKDANDVITMDPKDNCSKDANDTLLLQLTNTSAYYM
YLLLLLKSVVYFAIITCCLLRRTAFCCNGEKS

TCRδ2 (F283A/F290A/F291A (AAA)):

MDMRVPAQLLGLLLLWLSGARCMDYKDDDDKGGSETGAIELVPEHQTVPVSI
GVPATLRCSMKGEAIGNYYINWYRKTQGNTMTFIYREKDIYGPGFKDNFQGD
IDIAKNLAVLKILAPSERDEGSYYCACDTLGMGGEYTDKLIFGKGTRVTVEPR
SQPHTKPSVFVMKNGTNVACLVKEFYPKDIRINLVSSKKITEFDPAIVISPSGKY
NAVKLGKYEDSNSVTCSVQHDNKTVHSTDFEVKTDSTDHVKPKETENTKQPS
KSCHKPKAIVHTEKVNMMSLTVLGLRMLFAKTVAVNALLTAKLAAL

TCRδ2 (TMα (I254-S273)):

MDMRVPAQLLGLLLLWLSGARCMDYKDDDDKGGSETGAIELVPEHQTVPVSI
GVPATLRCSMKGEAIGNYYINWYRKTQGNTMTFIYREKDIYGPGFKDNFQGD
IDIAKNLAVLKILAPSERDEGSYYCACDTLGMGGEYTDKLIFGKGTRVTVEPR
SQPHTKPSVFVMKNGTNVACLVKEFYPKDIRINLVSSKKITEFDPAIVISPSGKY
NAVKLGKYEDSNSVTCSVQHDNKTVHSTDFEVKTDSTDHVKPKETENTKQPS
KSCHKPKAIVHTEKVNMMSLTVLGLRILLLKVAGFNLLMTLRLWSS

ALOD4:

MRGSHHHHHHGMASMTGGQQMGRDLYDDDDKDPCAKMTLDHYGAYVAQ
FDVSWDEFTFDQNGKEVLTHKTWEGSGKDKTAHYSTVIPLPPNSKNIKIVARE
ATGLAWEWWRTIINEQNVPLTNEIKVSIGGTTLYPTATISH

ALOD4-mutant:

MRGSHHHHHHGMASMTGGQQMGRDLYDDDDKDPCAKMTLDHYGAYVAQ
FDVSWDEFTFDQNGKEVLTHKTWEGSGKDKTAHYSTVIPLPPNSKNIKIVARE
ATGLAWEWWRTIINEQNVPLTNEIKVSIGAAAAAATATISH

ECD TCRδ1:

MDMRVPAQLLGLLLLWLSGARCMDYKDDDDKGGSETGAQKVTQAQSSVSM
PVRKAVTLNCLYETSWWSYYIFWYKQLPSKEMIFLIRQGSDEQNAKSGRYSV
NFKKAAKSVALTISALQLEDSAKYFCALGDPGGLNTDKLIFGKGTRVTVEPRS
QPHTKPSVFVMKNGTNVACLVKEFYPKDIRINLVSSKKITEFDPAIVISPSGKYN
AVKLGKYEDSNSVTCSVQHDNKTVHSTDFEVKTDSGSHHHHHH

ECD TCRγ5:

MDMRVPAQLLGLLLLWLSGARCMDYKDDDDKGGSETGSSNLEGGTKSVTRP
TRSSAEITCDLTVINAFYIHWYLHQEGKAPQRLLYYDVSNSKDVLESGLSPGK
YYTHTPRRWSWILILRNLIENDSGVYYCATWDRGNPKTHYYKKLFGSGTTLV
VTDKQLDADVSPKPTIFLPSIAETKLQKAGTYLCLLEKFFPDVIKIHWQEKKSN
TILGSQEGNTMKTNDTYMKFSWLTVPEKSLDKEHRCIVRHENNKNGVDQEIIF
PPIKTDVAAAWSHPQFEKGGGSGGGSGGSAWSHPQFEK

CD3γ-T2A-CD3ε-P2A-CD3δ-E2A-CD3ζ:

MEQGKGLAVLILAIILLQGTLAQSIKGNHLVKVYDYQEDGSVLLTCDAEAKNI
TWFKDGKMIGFLTEDKKKWNLGSNAKDPRGMYQCKGSQNKSKPLQVYYRM
CQNCIELNAATISGFLFAEIVSIFVLAVGVYFIAGQDGVRQSRASDKQTLLPND
QLYQPLKDREDDQYSHLQGNQLRRNGSGEGRGSLLTCGDVEENPGPMQSGT
HWRVLGLCLLSVGVWGQDGNEEMGGITQTPYKVSISGTTVILTCPQYPGSEIL
WQHNDKNIGGDEDDKNIGSDEDHLSLKEFSELEQSGYYVCYPRGSKPEDANF
YLYLRARVCENCMEMDVMSVATIVIVDICITGGLLLLVYYWSKNRKAKAKPV
TRGAGAGGRQRGQNKERPPPVPNPDYEPIRKGQRDLYSGLNQRRIGSGATNFS
LLKQAGDVEENPGPMEHSTFLSGLVLATLLSQVSPFKIPIEELEDRVFVNCNTSI
TWVEGTVGTLLSDITRLDLGKRILDPRGIYRCNGTDIYKDKESTVQVHYRMC
QSCVELDPATVAGIIVTDVIATLLLALGVFCFAGHETGRLSGAADTQALLRNDQ
VYQPLRDRDDAQYSHLGGNWARNKGSGQCTNYALLKLAGDVESNPGPMKW
KALFTAAILQAQLPITEAQSFGLLDPKLCYLLDGILFIYGVILTALFLRVKFSRSA
DAPAYQQGQNQLYNELNLGRREEYDVLDKRRGRDPEMGGKPQRRKNPQEGL
YNELQKDKMAEAYSEIGMKGERRRGKGHDGLYQGLSTATKDTYDALHMQA
LPPRAAAWSHPQFEKGGGSGGGSGGSAWSHPQFEK

TCRδ3:

MDMRVPAQLLGLLLLWLSGARCMDYKDDDDKGGSETDKVTQSSPDQTVAS
GSEVVLLCTYDTVYSNPDLFWYRIRPDYSFQFVFYGDNSRSEGADFTQGRFS
VKHILTQKAFHLVISPVRTEDSATYYCAFTGLGDTSHADKLIFGKGTRVTVEPR
SQPHTKPSVFVMKNGTNVACLVKEFYPKDIRINLVSSKKITEFDPAIVISPSGKY
NAVKLGKYEDSNSVTCSVQHDNKTVHSTDFEVKTDSTDHVKPKETENTKQPS
KSCHKPKAIVHTEKVNMMSLTVLGLRMLFAKTVAVNFLLTAKLFFL

TCRγ2:

MDMRVPAQLLGLLLLWLSGARCMDYKDDDDKGGSETGSSNLEGRTKSVIRQ
TGSSAEITCDLAEGSNGYIHWYLHQEGKAPQRLQYYDSYNSKVVLESGVSPG
KYYTYASTRNNLRLILRNLIENDFGVYYCATWDVKQNYYKKLFGSGTTLVVT
DKQLDADVSPKPTIFLPSIAETKLQKAGTYLCLLEKFFPDVIKIHWQEKKSNTI
LGSQEGNTMKTNDTYMKFSWLTVPEKSLDKEHRCIVRHENNKNGVDQEIIFP

PIKTDVITMDPKDNCSKDANDTLLLQLTNTSAYYMYLLLLLKSVVYFAIITCCL
LRRTAFCCNGEKS

TCRγ3:

MDMRVPAQLLGLLLLWLSGARCMDYKDDDDKGGSETGSSNLEGRTKSVTRQ
TGSSAEITCDLTVTNTFYIHWYLHQEGKAPQRLLYYDVSTARDVLESGLSPGK
YYTHTPRRWSWILRLQNLIENDSGVYYCATWDGYYYKKLFGSGTTLVVTDK
QLDADVSPKPTIFLPSIAETKLQKAGTYLCLLEKFFPDVIKIHWQEKKSNTILGS
QEGNTMKTNDTYMKFSWLTVPEKSLDKEHRCIVRHENNKNGVDQEIIFPPIKT
DVITMDPKDNCSKDANDTLLLQLTNTSAYYMYLLLLLKSVVYFAIITCCLLRR
TAFCCNGEKS

TCRγ4:

MDMRVPAQLLGLLLLWLSGARCMDYKDDDDKGGSETGSSNLEGRTKSVIRQ
TGSSAEITCDLAEGSTGYIHWYLHQEGKAPQRLLYYDSYTSSVVLESGISPGK
YDTYGSTRKNLRMILRNLIENDSGVYYCATWDGDYYKKLFGSGTTLVVTDK
QLDADVSPKPTIFLPSIAETKLQKAGTYLCLLEKFFPDVIKIHWQEKKSNTILGS
QEGNTMKTNDTYMKFSWLTVPEKSLDKEHRCIVRHENNKNGVDQEIIFPPIKT
DVITMDPKDNCSKDANDTLLLQLTNTSAYYMYLLLLLKSVVYFAIITCCLLRR
TAFCCNGEKS

TCRγ8:

MDMRVPAQLLGLLLLWLSGARCMDYKDDDDKGGSETGSSNLEGRTKSVTRP
TGSSAVITCDLPVENAVYTHWYLHQEGKAPQRLLYYDSYNSRVVLESGISREK
YHTYASTGKSLKFILENLIERDSGVYYCATWDSSKLFGSGTTLVVTDKQLDAD
VSPKPTIFLPSIAETKLQKAGTYLCLLEKFFPDVIKIHWQEKKSNTILGSQEGNT
MKTNDTYMKFSWLTVPEKSLDKEHRCIVRHENNKNGVDQEIIFPPIKTDVITM
DPKDNCSKDANDTLLLQLTNTSAYYMYLLLLLKSVVYFAIITCCLLRRTAFCC
NGEKS

Supplementary Figures

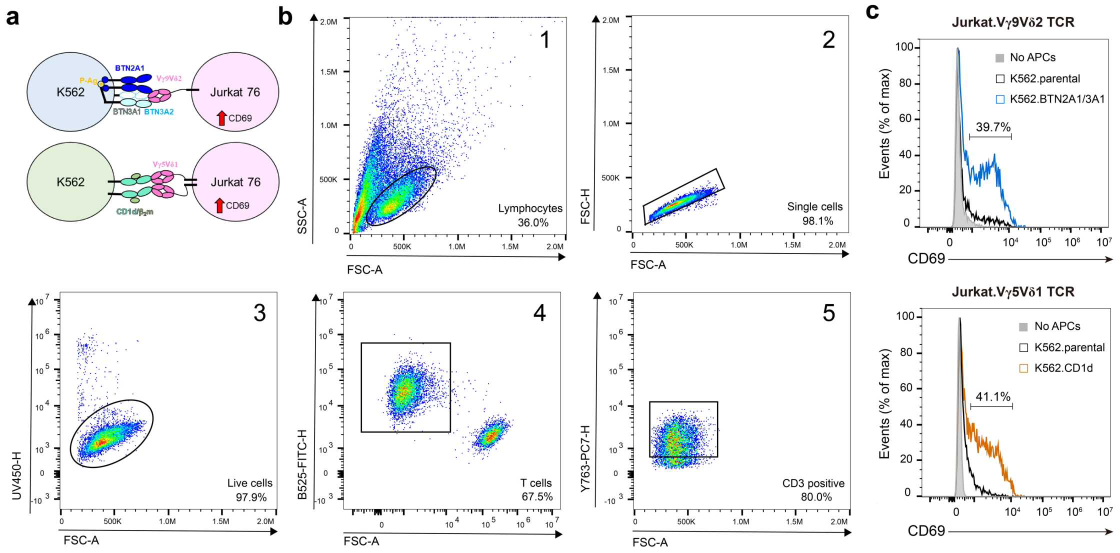

Supplementary Fig. 1 | Gating strategy used in the CD69 expression analysis and functional validation of Jurkat-76 cells stably expressing γδ TCR of interest. a, A schematic diagram of the T cell activation assay. An upregulation of CD69 expression occurs upon the activation of Jurkat-76 cells. P-Ag: phosphoantigens. The endogenous BTN3A2 that facilitates Vγ9Vδ2 TCR activation is indicated by dotted blue circle. **b**, Selection of $CD3^{+}$ Jurkat-76 cells through a series of five gates. The first gate is applied to remove cell debris, followed by the second gate to eliminate adhered cells. The third gate is applied to select live cells using DAPI. The fourth gate selects $mGreenLantern^{+}$ cells, and the fifth gate employs anti-CD3 antibodies conjugated with PE-Cyanine7 dye to select $CD3^{+}$ cells. **c**, Flow cytometry analysis of CD69 expression on G115 Vγ9Vδ2 (upper) or 9C2 Vγ5Vδ1 (lower) TCR transduced Jurkat-76 cells cocultured with K562 cells expressing BTN2A1/3A1 or CD1d or ZIM3–dCas9 (parental) or without K562 cells. Numbers in plots indicate percent of gated events. Data are representative of three independent experiments for all samples. APC: antigen-presenting cells.

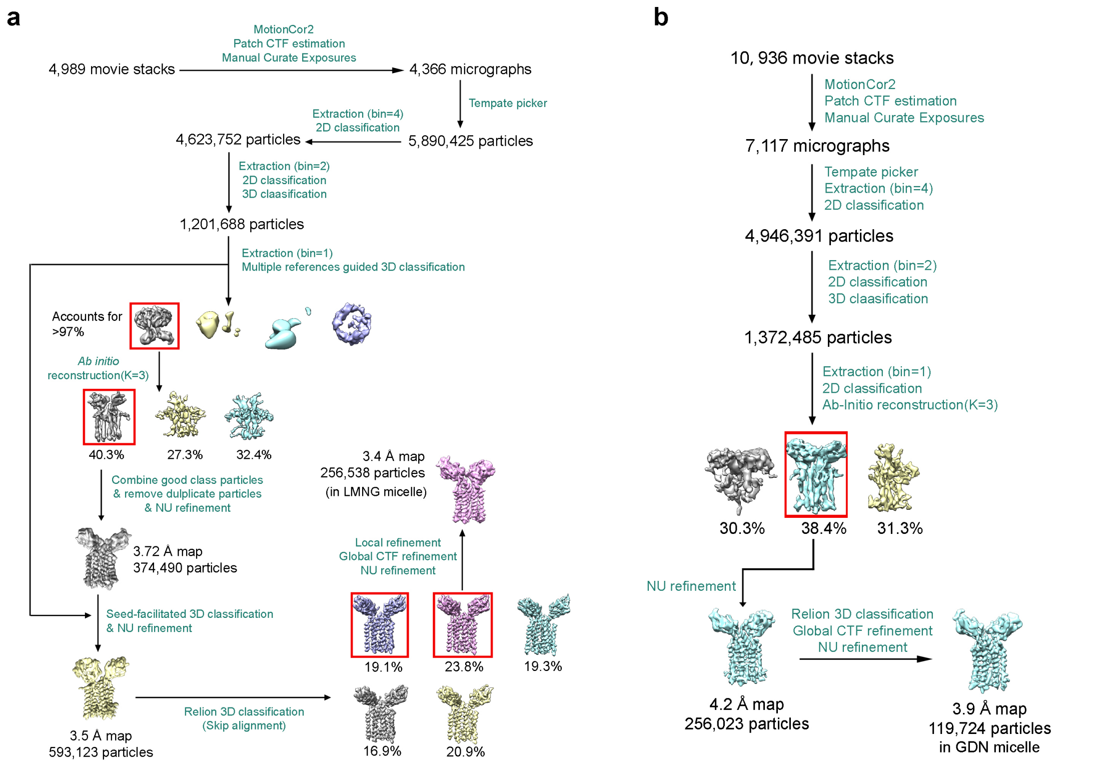

Supplementary Fig. 2 | The flowchart for cryo-EM data processing of the human Vγ9Vδ2 TCR–CD3 complex in LMNG or GDN detergent micelles. a, Summary of the data processing procedure of the Vγ9Vδ2 TCR–CD3 complex. **b**, Summary of the data processing procedure of the Vγ9Vδ2 TCR–CD3 complex in GDN detergent micelles. Details are described in Methods. NU: non-uniform.

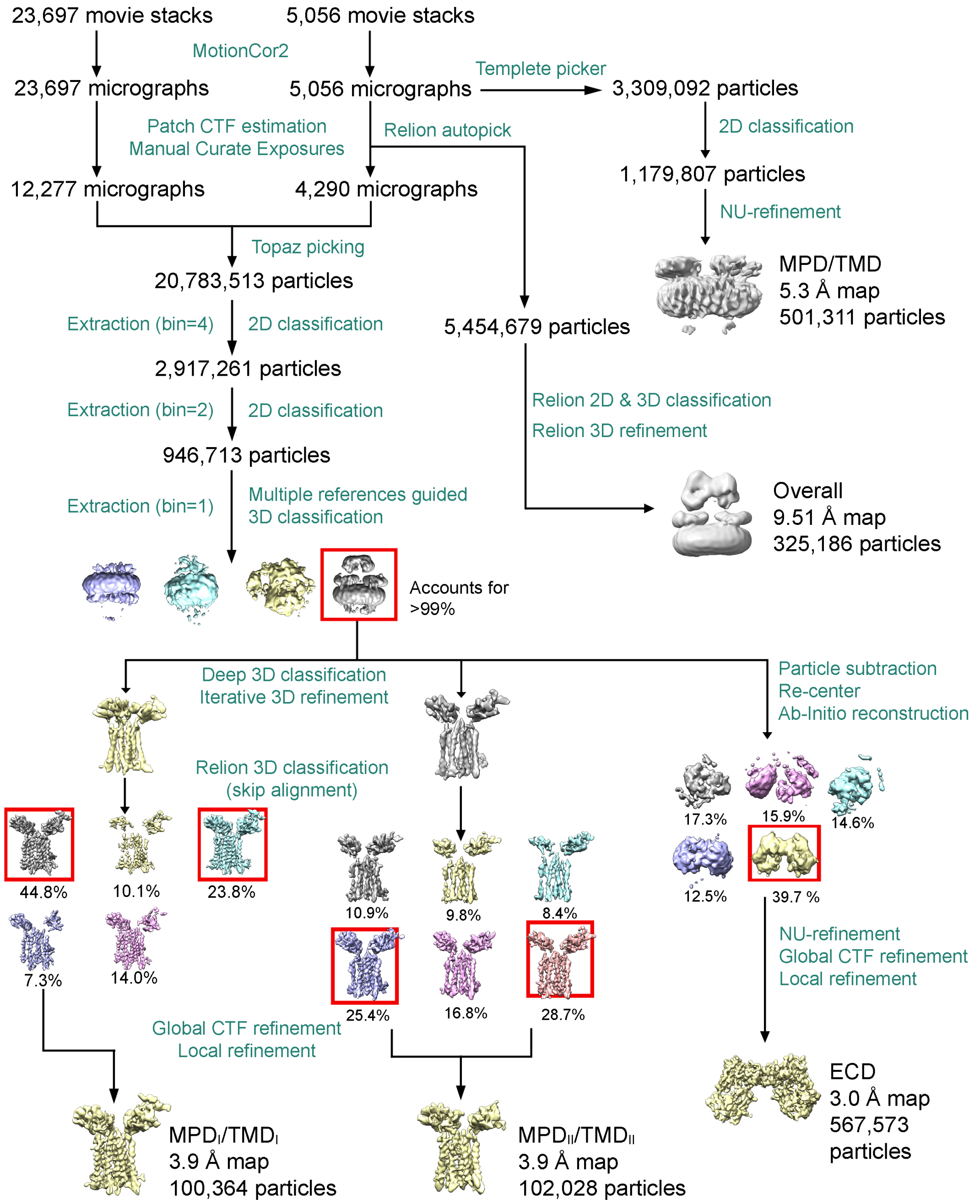

Supplementary Fig. 3 | The flowchart for cryo-EM data processing of the human Vγ5Vδ1 TCR–CD3 complex. Summary of the data processing procedure of the Vγ5Vδ1 TCR–CD3 complex. Details are described in Methods. NU: non-uniform.

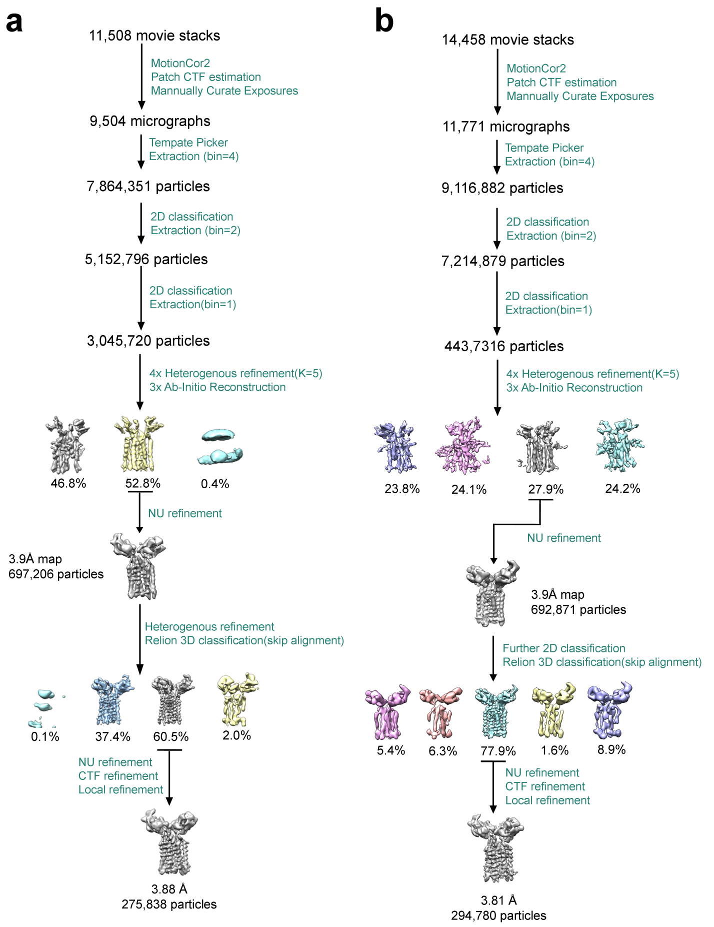

Supplementary Fig. 4 | The flowchart for cryo-EM data processing of the mutant Vγ9Vδ2 and mutant Vγ5Vδ1 TCR–CD3 complexes. a-c, Summary of the data processing procedure of the TMα Vγ9Vδ2 TCR–CD3 complex (**a**), AAA Vγ9Vδ2 TCR–CD3 complex (**b**), and EH Vγ5Vδ1 TCR–CD3 complex (**c**). Details are described in Methods. NU: non-uniform. TMα: replace TMδ (M273-L292) with TMα (I254-S273) in TCRδ2. AAA: F283A/F290A/F291A in TCRδ2. EH: Y106E/R120H in the Vγ5 domain.

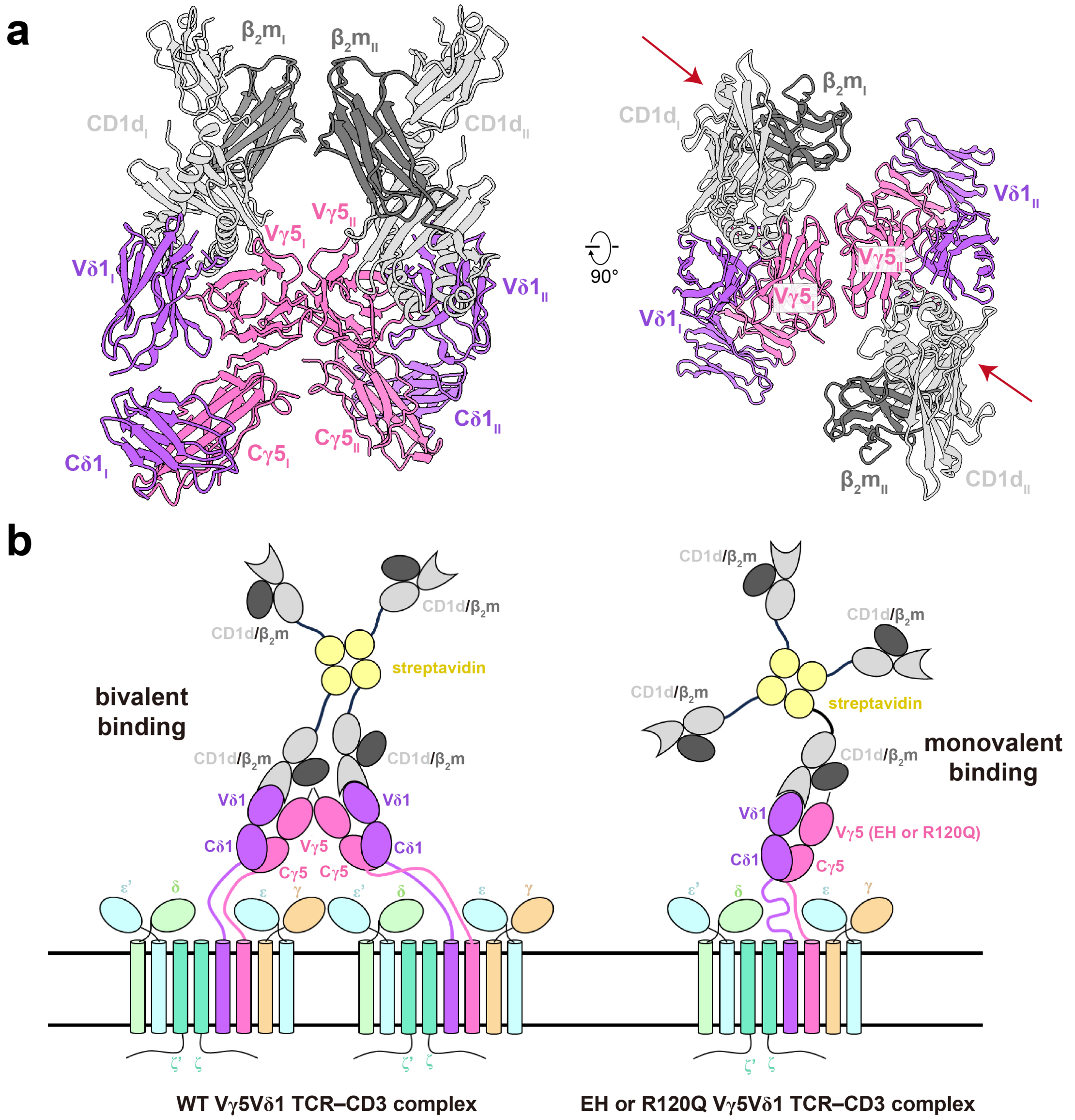

Supplementary Fig. 5 | Vγ5Vδ1 TCR–CD3 complex recognizes CD1d–β2m in a bivalent binding manner. a, The structural superimposition between the CD1d–β2m complex (gray, PDB: 4LHU) and the ECD of the Vγ5Vδ1 TCR–CD3 complex. The two docked CD1d–β2m molecules are distanced from each other and do not exhibit steric hindrance. The CD1d–β2m molecules are indicated by red arrows. Two perpendicular views are shown. **b**, A schematic diagram illustrates the binding of the dimeric Vγ5Vδ1 TCR-CD3 complex to CD1d-β2m at a ratio of 1:2. However, for the EH mutant Vγ5Vδ1 TCR-CD3 complex, the binding mode occurs at a ratio of 1:1.

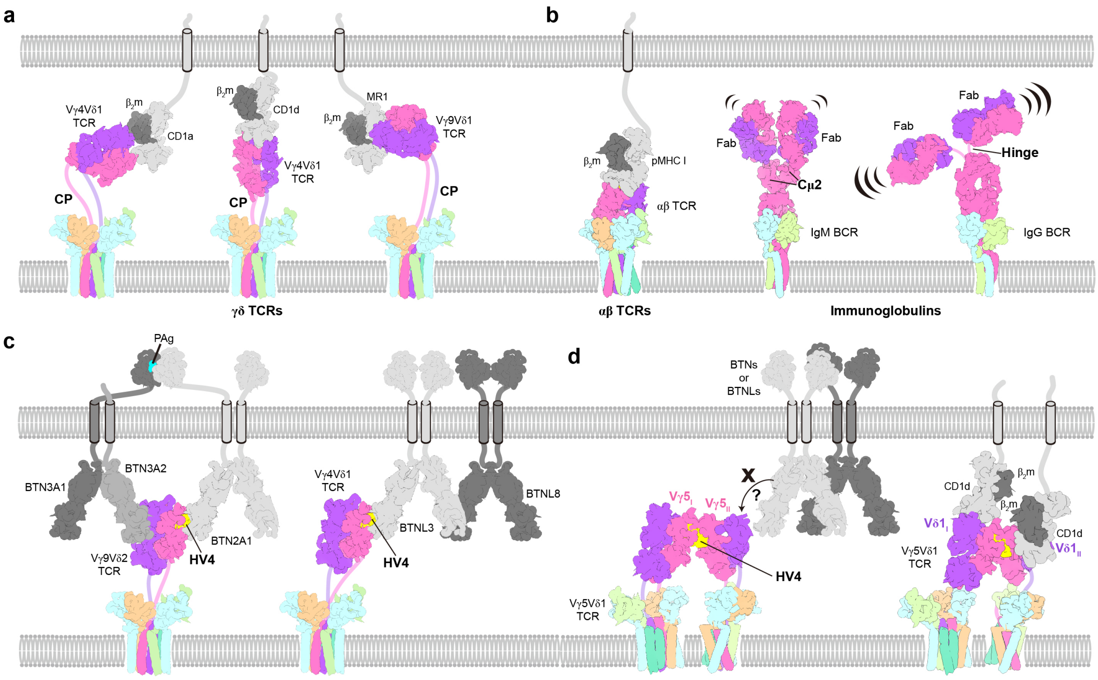

Supplementary Fig. 6 | Different ligand recognition modalities of the monomeric and dimeric γδ TCRs, αβ TCRs and BCRs. a, A schematic diagram showing the docking topologies among CO3 Vγ4Vδ1, DP10.7 Vγ4Vδ1, and G7 Vγ9Vδ1 TCR–CD3 with the ligands of CD1a–β2m, CD1d–β2m, and MR1–β2m. The exceptional flexibility of monomeric TCR ECDs and CPs permits the diverse docking topologies towards to their ligands. The figures were generated using the structures from Protein Data Bank (7RYN, 4MNG, and 6MWR). **b,** A schematic diagram showing the structural variation among αβ TCR–CD3 complex, IgM and IgG B cell receptors (BCRs). The γδ TCR–CD3 ECDs show similar conformational flexibility to BCR Fab domains in contrast to the rigid αβ TCR–CD3 ECDs. The figures were generated using the structures from Protein Data Bank (7PHR, 7XQ8, and 7WSO). **c,** A schematic diagram showing the docking topologies of BTN2A1/3A1/3A2 and BTNL3/L8 with monomeric G115 Vγ9Vδ2 and LES Vγ4Vδ1 TCR–CD3 complexes via the germline-encoded HV4 region (yellow), respectively. The figures were generated using the structures from Protein Data Bank (8DFW, 8IGT, 8DFX, 7RYL, and 1HXM). **d,** A schematic diagram showing the docking topology of dimeric Vγ5Vδ1 TCR–CD3 complex to CD1d–β2m. The dimeric γδ TCR–CD3 complex may not response to the BTNs or BTNLs or other potential ligands due to HV4 regions (yellow) being covered by Vγ domain dimerization. The figures were generated using the structures from Protein Data Bank (4F9P, 8DFX, and 4LHU).

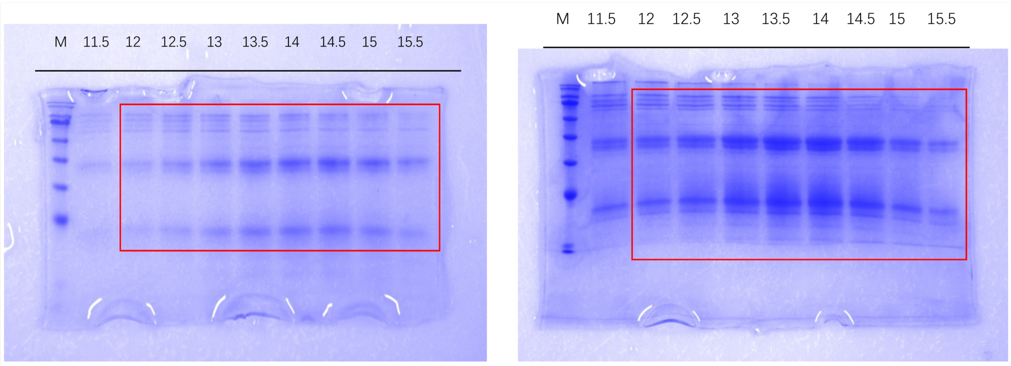

Supplementary Fig. 7 | Uncropped images for the Extended Data Figure 1c.

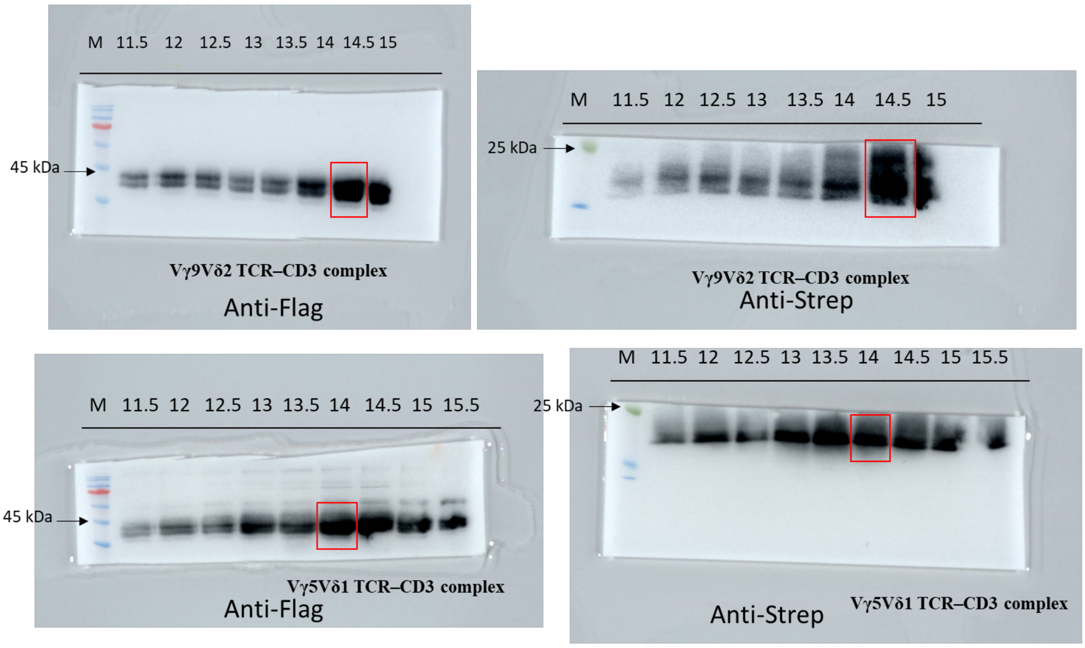

Supplementary Fig. 8 | Uncropped images for the Extended Data Figure 1d.

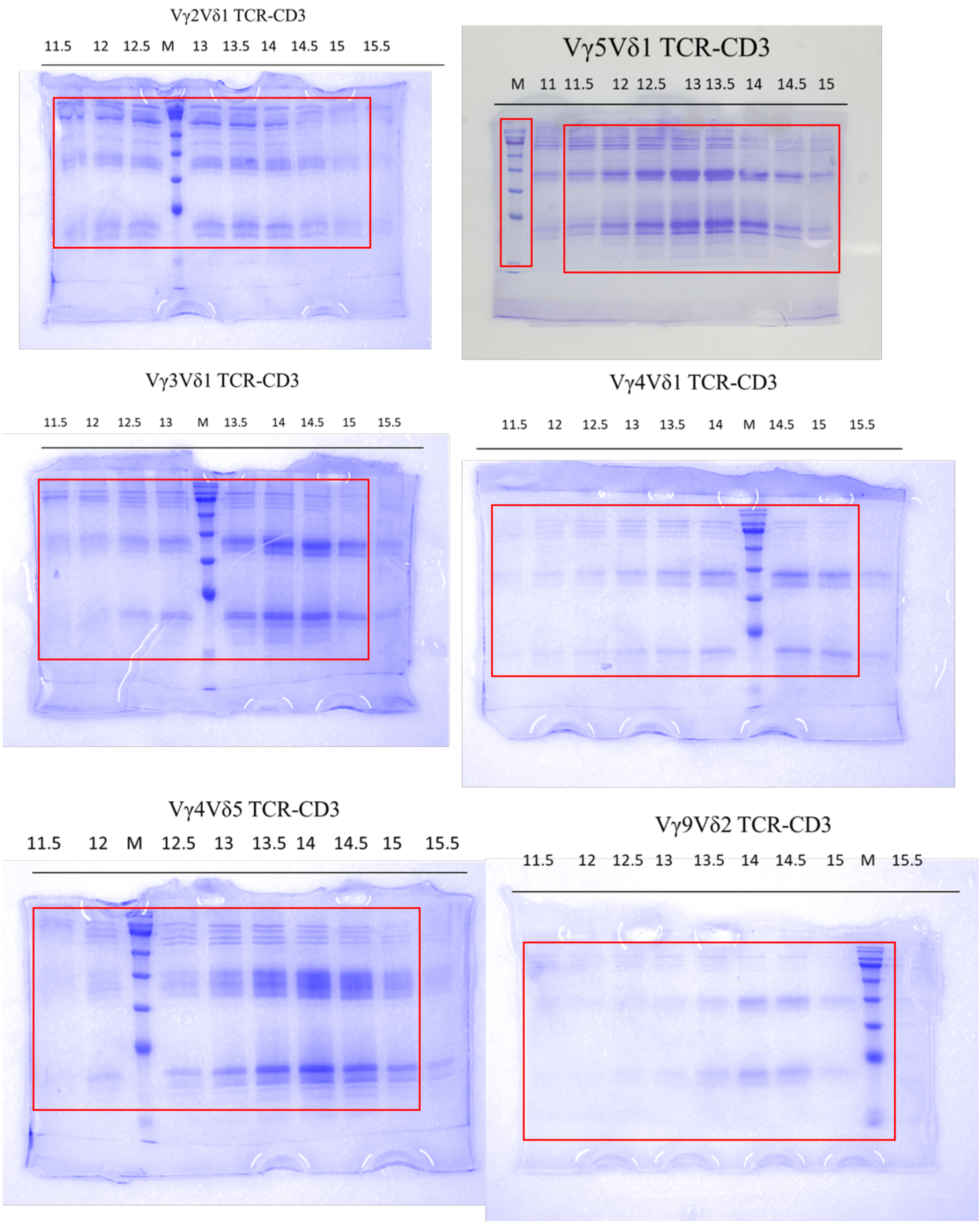

Supplementary Fig. 9 | Uncropped images for the Extended Data Figure 8b.

Supplementary Table S1 | The interactions within the Vγ5$_{I}$–Vγ5$_{II}$ interface.

Vγ5$_{I}$	Vγ5$_{II}$	Interaction
R120	S92	Hydrogen bond
R120	T107/O	Hydrogen bond
R120	D94	Salt bridge
S92	R120	Hydrogen bond
T107/O	R120	Hydrogen bond
D94	R120	Salt bridge
R120	Y106	Cation-π
Y106	R120	Cation-π
Y106	Y106	π-π
E57	H108	Hydrogen bond
H108	E57	Hydrogen bond
H108	I118	Van der Waals effect
I118	H108	Van der Waals effect
