# Supplementary material for: Structures of human γδ T cell receptor–CD3 complex
Source: Nature. 2024 Apr 24;630(8015):222–9. doi: 10.1038/s41586-024-07439-4 (PMC11153141; doi:10.1038/s41586-024-07439-4)
Supplement: Supplementary file 2 — Reporting Summary [file 41586_2024_7439_MOESM2_ESM.pdf]

nature portfolio

Corresponding author(s): Qiang Zhou, Qiang Su

Last updated by author(s): Apr 5, 2024

Reporting Summary

Nature Portfolio wishes to improve the reproducibility of the work that we publish. This form provides structure for consistency and transparency in reporting. For further information on Nature Portfolio policies, see our Editorial Policies and the Editorial Policy Checklist.

Statistics

For all statistical analyses, confirm that the following items are present in the figure legend, table legend, main text, or Methods section.

n/a	Confirmed	
☐	☒	The exact sample size (*n*) for each experimental group/condition, given as a discrete number and unit of measurement
☐	☒	A statement on whether measurements were taken from distinct samples or whether the same sample was measured repeatedly
☐	☒	The statistical test(s) used AND whether they are one- or two-sided<br>*Only common tests should be described solely by name; describe more complex techniques in the Methods section.*
☒	☐	A description of all covariates tested
☒	☐	A description of any assumptions or corrections, such as tests of normality and adjustment for multiple comparisons
☐	☒	A full description of the statistical parameters including central tendency (e.g. means) or other basic estimates (e.g. regression coefficient) AND variation (e.g. standard deviation) or associated estimates of uncertainty (e.g. confidence intervals)
☐	☒	For null hypothesis testing, the test statistic (e.g. *F*, *t*, *r*) with confidence intervals, effect sizes, degrees of freedom and *P* value noted<br>*Give P values as exact values whenever suitable.*
☒	☐	For Bayesian analysis, information on the choice of priors and Markov chain Monte Carlo settings
☒	☐	For hierarchical and complex designs, identification of the appropriate level for tests and full reporting of outcomes
☒	☐	Estimates of effect sizes (e.g. Cohen's *d*, Pearson's *r*), indicating how they were calculated

Our web collection on statistics for biologists contains articles on many of the points above.

Software and code

Policy information about availability of computer code

Data collection	EPU 2
Data analysis	Relion 3.1, Coot 0.8.9.1, Chimera 1.11.2, cryoSPARC v4, ChimeraX 1.1, Phenix 1.18.1, GraphPad Prism 9, FlowJo 10.6.2, LAS X, UCSF pyem 0.5 software suite.

For manuscripts utilizing custom algorithms or software that are central to the research but not yet described in published literature, software must be made available to editors and reviewers. We strongly encourage code deposition in a community repository (e.g. GitHub). See the Nature Portfolio guidelines for submitting code & software for further information.

Data

Policy information about availability of data

All manuscripts must include a data availability statement. This statement should provide the following information, where applicable:

- Accession codes, unique identifiers, or web links for publicly available datasets
- A description of any restrictions on data availability
- For clinical datasets or third party data, please ensure that the statement adheres to our policy

The atomic coordinates for Vγ9Vδ2 and Vγ5Vδ1 TCR–CD3 complexes have been deposited in the Protein Data Bank (PDB) with the accession codes 8JC0 (WT/LMNG), 8YC0 (WT/GDN), 8WYI (TMα), 8WY0 (AAA), 8JCB (Overall), 8JBV (ECD), and 8WXE (EH), respectively. For Vγ9Vδ2 TCR–CD3 complexes, the cryo-EM maps sharpened by DeepEMhancer have been deposited in the Electron Microscopy Data Bank (EMDB) with the accession codes EMD-36149 (WT/LMNG), EMD-39128

(WT/GDN), EMD-37929 (TMα), EMD-37914 (AAA), respectively. The cryo-EM maps sharpened using B-factor have been deposited in the EMDB with the accession codes EMD-39363 (WT/LMNG), EMD-39359 (WT/GDN), EMD-39366 (TMα), EMD-39364 (AAA), respectively. For Vγ5Vδ1 TCR–CD3 complexes, the cryo-EM maps sharpened by DeepEMhancer of have been deposited in the EMDB with the accession codes EMD-36152 (MPDI/TMDI), EMD-36153 (MPDII/TMDII), EMD-36147 (ECD), and EMD-37904 (EH). The cryo-EM maps sharpened using B-factor have been deposited in the EMDB with the accession codes EMD-36156 (Overall), EMD-36155 (MPD/TMD), EMD-39361 (MPDI/TMDI), EMD-39362 (MPDII/TMDII), EMD-39368 (ECD), and EMD-39367 (EH). To prepare figures, we used structural information from the PDB with the accession codes 7FJD, 1HXM, 7FJE, 4LHU, 7RYN, 4MNG, 6MWR, 7PHR, 7XQ8, 7WSO, 8DFW, 8IGT, 8DFX, 7RYL, and 4F9P. All data are available in the manuscript and the Supplementary Information. Source data are provided with this paper. All materials are available from the corresponding authors upon reasonable request.

Research involving human participants, their data, or biological material

Policy information about studies with human participants or human data. See also policy information about sex, gender (identity/presentation), and sexual orientation and race, ethnicity and racism.

Reporting on sex and gender	n/a
Reporting on race, ethnicity, or other socially relevant groupings	n/a
Population characteristics	n/a
Recruitment	n/a
Ethics oversight	n/a

Note that full information on the approval of the study protocol must also be provided in the manuscript.

Field-specific reporting

Please select the one below that is the best fit for your research. If you are not sure, read the appropriate sections before making your selection.

☒ Life sciences ☐ Behavioural & social sciences ☐ Ecological, evolutionary & environmental sciences

For a reference copy of the document with all sections, see nature.com/documents/nr-reporting-summary-flat.pdf

Life sciences study design

All studies must disclose on these points even when the disclosure is negative.

Sample size	Sample sizes for the Cryo-EM datasets were determined by the need to obtain structures with sufficient resolution. Sample sizes for all cell-based assay was determined by conclusive results we could get. No calculations were performed to determine sample sizes; however, the addition of more data did not alter conclusions from this study.
Data exclusions	Some data points were not successful and excluded from the assays.
Replication	Experiment was repeated at least twice and the results were successfully reproduced. The number of biological and technical replicates were indicated in the figure legends.
Randomization	For the structural studies, during the 3D refinement process, the particle images were randomly divided into two separate groups. During 2D classification, each particles assigned to a random class with a random orientation. For the cell-based assay, the cells used was allocated randomly.
Blinding	For all experiments, there are both negative and positive controls and all the results were obtained in parallel using the same setting, and each treatment was assigned to a number during the experiment.

Reporting for specific materials, systems and methods

We require information from authors about some types of materials, experimental systems and methods used in many studies. Here, indicate whether each material, system or method listed is relevant to your study. If you are not sure if a list item applies to your research, read the appropriate section before selecting a response.

Materials & experimental systems

n/a	Involved in the study	
☐	☒	Antibodies
☐	☒	Eukaryotic cell lines
☒	☐	Palaeontology and archaeology
☒	☐	Animals and other organisms
☒	☐	Clinical data
☒	☐	Dual use research of concern
☒	☐	Plants

Methods

n/a	Involved in the study	
☒	☐	ChIP-seq
☐	☒	Flow cytometry
☒	☐	MRI-based neuroimaging

Antibodies

Antibodies used

Primary antibodies: mouse monoclonal antibodies against Flag tag (Cat: CW0287M, CWBIO, clone name: F-tag-01), Strep tag (Cat: ab76949, Abcam, Polyclonal). Secondary antibodies: Goat Anti-Mouse IgG (Cat: CW0102, CWBIO). Flow cytometry antibodies: Anti-human CD69-APC (allophycocyanin) (Sino Biological Inc; Cat: 11150-MM06-A, clone name: #06) and CD3-PE (phycoerythrin)-Cyanine7 dye antibodies (BD Pharmingen; Cat: 552127, clone name: SP34-2). anti-CD3 (Invitrogen; Cat: 16-0037-81; clone name: OKT3) and anti-CD28 antibodies (Invitrogen; Cat: 16-0289-81; clone name: CD28.2), anti-Flag antibodies (ABclonal; Cat: AE116; clone name: AMC0382-PE)

Validation

All antibodies are commercially available and validated by the manufacturer to demonstrate its ability to detect corresponding target. None were independently validated by us.

Eukaryotic cell lines

Policy information about cell lines and Sex and Gender in Research

Cell line source(s)

The Expi293F cell were purchased from Thermo Fisher Scientific Inc.; (Code No. A14527CN). Lenti-X 293T was from TaKaRa (Clontech; Code No.632180). The parental K562 cells were from ATCC® CCL-243™. The parental Jurkat 76 cells were from Chenqi Xu Lab at Shanghai Institute of Biochemistry. Transduced K562 and Jurkat cell lines were generated and validated in house.

Authentication

Jurkat76 cell lines were checked for TCR expression by staining with antiCD3 and anti γδ TCR antibodies. The other cell lines cell lines used were not authenticated.

Mycoplasma contamination

All of the cell lines in this study were not tested for mycoplasma contamination.

Commonly misidentified lines
(See ICLAC register)

There is no commonly misidentified cell lines used in this study.

Flow Cytometry

Plots

Confirm that:

☒ The axis labels state the marker and fluorochrome used (e.g. CD4-FITC).

☒ The axis scales are clearly visible. Include numbers along axes only for bottom left plot of group (a 'group' is an analysis of identical markers).

☒ All plots are contour plots with outliers or pseudocolor plots.

☒ A numerical value for number of cells or percentage (with statistics) is provided.

Methodology

Sample preparation

The Jurkat and K562 cells were harvested and washed twice in fluorescence-activated cell sorting (FACS) buffer consisting of phosphate-buffered saline (PBS) supplemented with 2% (v/v) fetal bovine serum (FBS). The cells were then incubated with anti-human CD69-APC (allophycocyanin) (Sino Biological Inc) and CD3-PE (phycoerythrin)-Cyanine7 dye antibodies (BD Pharmingen) at a dilution of 1:500 for 30 minutes on ice. Following this, the cells were washed again in FACS buffer. Before the FACS analysis, 4',6-diamidino-2-phenylindole (DAPI) (Solarbio) was added into the samples at a final concentration of 0.1 μg/mL.

Instrument

Samples were acquired on Cyto-FLEX LX -5L2 (Beckman).

Software

Resulting data was analyzed in FlowJo (version 10.8.1; Treestar & BD Biosciences).

Cell population abundance

Cells were expanded as described before they were analyzed after sorting. Sorting and post-sort analyses are shown in

Cell population abundance	Extended Data Fig. 2b, including the gate that was used to determine abundance.
Gating strategy	T cell activation assays: FSC-A/SSC-A > Singlets using FSC-A/FSC-H > Viable cells using FSC-A/DAPI > mGreenLantern positive cells using FSC-A/FITC > CD3 positive cells using FSC-A/PEcy7. The gate strategy was shown in Supplementary Fig. 1b.

☒ Tick this box to confirm that a figure exemplifying the gating strategy is provided in the Supplementary Information.
